# Three Dimensional Printing of Multiscale Carbon Fiber-Reinforced Polymer Composites Containing Graphene or Carbon Nanotubes

**DOI:** 10.3390/nano12122064

**Published:** 2022-06-15

**Authors:** Sara Residori, Sithiprumnea Dul, Alessandro Pegoretti, Luca Fambri, Nicola M. Pugno

**Affiliations:** 1 Laboratory for Bioinspired, Bionic, Nano, Meta Materials & Mechanics, Department of Civil, Environmental and Mechanical Engineering, University of Trento, Via Mesiano, 77, 38123 Trento, Italy; sara.residori@unitn.it; 2 Department of Industrial Engineering and INSTM Research Unit, University of Trento, Via Sommarive, 9, 38123 Trento, Italy; sithiprumnea_dul@yahoo.com (S.D.); alessandro.pegoretti@unitn.it (A.P.); 3 School of Engineering and Material Science, Queen Mary University of London, Mile End Road, London E1 4NS, UK

**Keywords:** multiscale composites, mechanical properties, fused filament fabrication, mechanical properties, electrical conductivity, selective parameters

## Abstract

Three-dimensional printing offers a promising, challenging opportunity to manufacture component parts with ad hoc designed composite materials. In this study, the novelty of the research is the production of multiscale composites by means of a solvent-free process based on melt compounding of acrylonitrile–butadiene–styrene (ABS), with various amounts of microfillers, i.e., milled (M) carbon fibers (CFs) and nanofillers, i.e., carbon nanotubes (CNTs) or graphene nanoplatelets (GNPs). The compounded materials were processed into compression molded sheets and into extruded filaments. The latter were then used to print fused filament fabrication (FFF) specimens. The multiscale addition of the microfillers inside the ABS matrix caused a notable increase in rigidity and a slight increase in strength. However, it also brought about a significant reduction of the strain at break. Importantly, GNPs addition had a good impact on the rigidity of the materials, whereas CNTs favored/improved the composites’ electrical conductivity. In particular, the addition of this nanofiller was very effective in improving the electrical conductivity compared to pure ABS and micro composites, even with the lowest CNT content. However, the filament extrusion and FFF process led to the creation of voids within the structure, causing a significant loss of mechanical properties and a slight improvement of the electrical conductivity of the printed multiscale composites. Selective parameters have been presented for the comparison and selection of compositions of multiscale nanocomposites.

## 1. Introduction

The expansion of the use of thermoplastic composite materials with different scale reinforcements for additive manufacturing has recently sparked considerable interest, because embedded multiscale particles offer the potential to improve various properties of 3D-printed polymers [1,2,3], such as mechanical properties [4,5], thermal stability [6,7], and electrical conductivity [8,9]. In particular, the investigation of materials with both micro- and nano-reinforcements has been shown to represent a further potential means to expand the production of parts by fused filament fabrication (FFF), which is a widely used 3D printing technology [10].

In the last decade, intensive research efforts have been focused on the progressive development of new polymeric formulations suitable for additive manufacturing. Indeed, the dispersion of particles at various length scales within plastic matrices allows the production of elements that are useful in every field of research and industry. For example, carbon-based particles render polymeric composites advantageous, since their introduction in polymers results in a stiffness increase, weight reduction, and higher corrosion resistance [11]. For instance, Zaldivar et al. [12] showed that the infiltration into 3D-printed acrylonitrile–butadiene–styrene (ABS) of epoxy resin containing 10 wt.% of milled carbon fibers increased the flexural modulus by 76% with respect to the neat material. Similarly, another study showed that the use of 1.5 wt.% of ZrB2 microparticle reinforcements in the ABS matrix resulted in an increase of strength and strain at break of about 13% and 82%, respectively [13]. Zhang et al. [14] also produced in 3D printing at +45° specimens of ABS and ABS composites containing 15 wt.% of short carbon fibers (CFs) with tensile strengths of 24 MPa and 29 MPa and moduli of 2.1 GPa and 2.8 GPa, respectively. The porosities of these 3D-printed specimens were estimated at about 0.5% and 8.5% for ABS and ABS/CF, respectively. Tekinalp et al. [15] reported that 40 wt.% short CF (3.2 mm length) in ABS high oriented fiber composites imparted strength and elastic modulus values of about 67 MPa and 13.8 GPa. The effect of milled carbon fiber, MCF (length of 100–150 microns; diameter of 7 microns), up to 15 wt.% was studied by Ning et al. [16]; they showed a maximum Young’s modulus (2.5 GPa) and strength (about 43 MPa) for fused deposition modeling (FDM) specimens with 7.5% of MCF. Recently, Adeniram et al. produced similar ABS micro composites by additive manufacturing with MCF content in the range of 10–30%, showing a higher toughness for the composition at 20% and a corresponding tensile modulus and strength of 3.7 GPa and 35 MPa, respectively [17].

As for nanocomposites, the dispersion of conductive nanoparticles in a polymer matrix makes it possible to produce 3D-printed components for various applications such as electronic sensors [18,19,20], circuits [21], and micro-batteries [22]. For example, the production of flexible, high-conductive poly(vinylidene fluoride) (PVDF) was reached with the addition of up to 15 wt.% of multi-walled carbon nanotube (MWCNT) [23]. The thermal expansion of PVDF was minimized by the presence of MWCNT. A direct correlation between MWCNT concentration and change in resistance was observed, with a larger response generated with reduced MWCNT loads. In addition, other studies report that a small amount of CNTs (less than 1 vol.%) in polylactic acid (PLA) matrix [24] or larger amounts (up to 8 wt.%) in ABS [25] improved the electrical conductivity. Also, graphene nanoplatelets (GNPs) in the amount of 4 wt.% were dispersed in ABS, leading to an increase in the elastic modulus and thermal stability but a concurrent decrease in both stress and strain at break [26]. Other studies have reported a significant increase in the values of interfacial shear strength in the matrices of nano-modified polypropylene (PP) with graphene nanoparticle fillers (up to a factor of about 6 for a content of 7 wt.% of xGnP) compared to pure PP [27]. Moreover, a hybrid composition at 6 wt.% of ABS nanocomposites was also studied in order to optimize the relative effect of GNPs for the stiffening increase and CNTs for electrical conductivity, with a proper balance between the processability and the properties [28]; in particular, a maximum 3 wt.% of CNT was suggested to avoid a too low reduction of melt flow index.

In this study, we investigated the efficacy of directly dispersing reinforcements on a different scale in the ABS matrix to produce multiscale composite filaments with a standard diameter of about 1.75 mm for the FFF process. Multiscale composite filaments were produced using common industrial processing techniques such as the internal mixer and the twin-screw extruder to mix polymer pellets with micro- and nanofillers. Mechanical and electrical characterization were performed on the compression-molded samples. Successively, selected filaments were used to feed an FFF 3D printer to highlight the effects of the multiscale reinforcements on composite material properties.

## 2. Materials and Methods

### 2.1. Materials

Acrylonitrile–butadiene–styrene (ABS) polymer (tradename Sinkral^®^ PD L322) was supplied by Versalis S.p.A. (Mantova, Italy) in the format of white pellets. The characteristics of the material were a density of 1.04 g/cm^3^, a melt flow index of 23 g/10 min (220 °C/10 kg), a flexural strength of 70 MPa, a tensile strength of 45 MPa, and an electrical resistivity of 10^14^ Ω·cm, as declared by the manufacturer [29]. Due to water absorption (0.3% at 23 °C/24 h), the ABS pellets were dried under a vacuum at 80 °C for at least 12 h before processing. Milled carbon fibers (MCFs) by Zoltek Europe (Nyergesujfalu, Hungary), multi-wall carbon nanotubes (CNTs) by Nanocyl S.A. (Sambreville, Belgium), and graphene nanoplatelets (GNPs) by XG Sciences (East Lansing, MI, USA) were selected for this work. Details of their sizes and physical properties, according to the manufacturer’s datasheet are presented in Table 1.

### 2.2. Materials Processing and Sample Preparation

Various multiscale carbonaceous composites based on microscale filler MCFs at a high percentage (10, 20, and 30 wt.%) and two different nanoscale fillers at a lower percentage, between 1 and 3 wt.% for CNTs or 3 and 9 wt.% for GNPs, were compounded, as detailed in Table 2. In particular, the filler ratio of CNT/MCF and GNP/MCF in the range of 0.03–0.30 and 0.15–0.45, respectively, were fixed. All materials were processed by compression molding, and then selected compositions were used for filament extrusion and 3D printing.

#### 2.2.1. Compounding

All composites in this study were processed by a Thermo-Haake Polylab Rheomix 600 (Thermo-Haake, Karlsruhe, Germany) counter-rotating internal mixer at 210 °C, with a rotor speed of 90 rpm for a total time of 15 min. For microfiber composites, ABS was firstly heated for 4 min at 210 °C, followed by the addition of MCFs at various amounts (10, 20, and 30 wt.%). For two different multiscale composites, various amounts of CNTs (1, 2, and 3 wt.%) or GNPs (3, 6, and 9 wt.%) were added after 6 min. Over the next 9 min of compounding, the mixer provided a constant torque value, which could indicate the leveling of the filler’s dispersion and the absence of degradation of the matrix. Neat ABS was also processed under the same conditions as a reference material. For each composition, about 50 g was produced. The details of the formulations and the processing techniques for the selected compositions are summarized in Table 2.

#### 2.2.2. Compression Molding (CM)

The compounded materials were heated at 210 °C and shaped by using a hydraulic laboratory press (Carver, Wabash, IN, USA) under a pressure of 3.9 MPa applied for 10 min, and then were cooled at 20 °C/min. Square plates with dimensions of 160 mm × 160 mm × 1.2 mm were produced, and handlebar samples were die punched in the desired shape for mechanical and electrical characterization. The code CM will be used henceforth to identify the samples prepared by compression molding.

#### 2.2.3. Filament Extrusion

The selected compounded materials, i.e., ABS and eight compositions (Table 2), were milled by a using grinder IKA M20 Universal mill (IKA^®^-Werke GmbH & Co. KG, Staufen, Germany) in order to obtain suitable powder for extrusion. The production of the filaments required for the 3D-printing process was carried out by a Thermo Haake PTW16 (Thermo Haake, Karlsruhe, Germany) intermeshing co-rotating twin-screw extruder. The processing temperature was gradually increased from 150 °C (zone 1) to 210 °C (zone 2), to 220 °C (zone 3), to 230 °C (zone 4), and to 240 °C (zone 5—rod die). The screw rotation speed was fixed at 10 rpm and the collection rate was properly adjusted to collect extruded filaments with a final diameter equal to 1.75 ± 0.10 mm, as an average of at least 20 measurements. During the extrusion process, the diameter was measured every 20 cm in order to guarantee an adequate geometry for feeding the 3D printer. Generally, the first two meters of extruded filaments were discarded, and then for each composition at least four meters of regular filament were produced.

**Figure 1:**
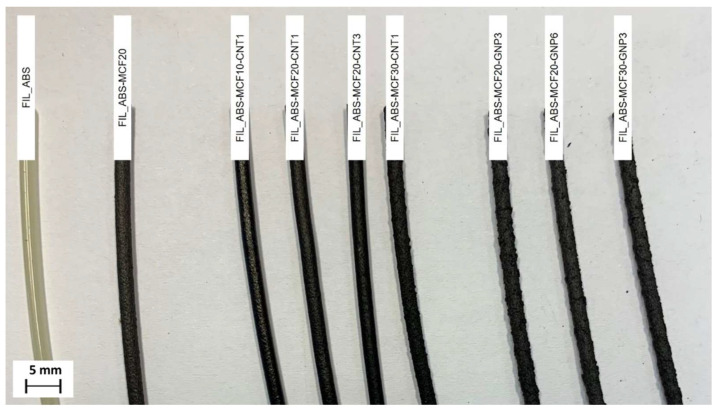
Extruded filaments of neat ABS, ABS/MCF, ABS/MCF/CNT, and ABS/MCF/GNP composites. Each composition is detailed in the inset.

The code FIL_ followed by the material composition (in wt.%) will henceforth be used to identify the extruded filaments, as shown in Figure 1.

#### 2.2.4. FFF Printed Samples Preparation

**Figure 2:**
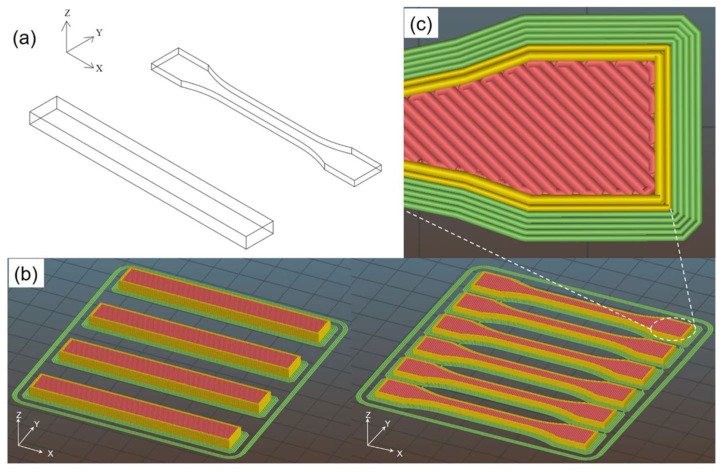
Schematic of the 1BA dumbbell and parallelepiped specimen (80 mm × 10 mm × 3.8 mm) (**a**), the arrangement of 3D-printed samples in Sil3r software (**b**), and detail of infill pattern (**c**).

The extruded filaments were used to feed a Sharebot Next Generation desktop 3D printer (Sharebot NG, Nibionno, LC, Italy). The manufactured products are schematically illustrated in Figure 2: dumbbell and parallelepiped samples were constructed along with the horizontal orientation with a filament printing angle of ±45°.

The dimensions and the processing parameters of the FFF samples are summarized in Table 3. The axis X is the main direction of filament deposition, Y is the direction of the sample width, and Z is the direction of the overlapping layers. The design of the concentric fill type and the maximum fill percentage was drafted by the Slic3r software, with specific general printing parameters (no raft; nozzle diameter of 0.56 mm; layer height of 0.20 mm; nozzle temperature of 250 °C; bed temperature of 110 °C; deposition rate fixed at 40 mm/s). A higher nozzle temperature, 280 °C, was required for ABS-MCF20-GNP6 and ABS-MCF30-GNP3 in order to avoid undesired clogging phenomena, probably associated with the combination of graphene nanoplatelets in high content MCF composites (see the high value of the total composite aspect ratio for these compositions in Table 4).

The code 3D will henceforth be used to identify a 3D-printed sample.

### 2.3. Testing Techniques

#### 2.3.1. Melt Flow Index

Melt flow index (MFI) measurement was performed using a Kayeness Co. model 4003DE capillary rheometer (Morgantown, PA, USA), according to ASTM D1238 standard (procedure A). For ABS and each composite, approximately 5 g of chopped CM material was tested at 220 °C under an applied load of 10 kg. Before the test, the material was pre-heated and compacted for 5 min. At least five measurements for each composition were considered (details in the Supplementary Materials, Table S2).

#### 2.3.2. Density Measurements

The density of bulk CM and 3D specimens *ρ_m_* was measured by using the analytical balance Gibertini E42 (Gibertini, Novate Milanese, MI, Italy) at 23 °C in accordance with ASTM D792 following Equation (1):

(1) $$\rho_{m}=\frac{m_{air}\cdot \rho_{ethanol}}{m_{air}-m_{ethanol}}$$

where *m_air_* is the air mass, *ρ_ethanol_* is the relative density of the ethanol, and *m_ethanol_* is the ethanol mass. The results represent the average of at least three measurements.

The theoretical density, *ρ_c_*, was calculated according to Equation (2) as:

(2) $$\frac{1}{\rho_{c}}=\overset{n}{\underset{i=1}{\sum }}\frac{W_{i}}{\rho_{i}}$$

where *W_i_* is the weight of the single composite content and *ρ_i_* is its relative density. The volume % of voids, *V_v_*, due to the production process was estimated from Equation (3) as:

(3) $$V_{v}=\frac{\rho_{c}-\rho_{m}}{\rho_{c}}\times 100$$

where *ρ_c_* and *ρ_m_* are the theoretical and the experimental density, respectively.

#### 2.3.3. Scanning Electron Microscopy (SEM)

Morphological analysis of composite products (compression-molded plates, extruded filaments, and 3D-printed samples) was performed on the cross-section obtained by a fragile fracture in liquid nitrogen. Specimens were observed by a Carl Zeiss AG Supra 40 field-emission scanning electron microscope (FESEM) (Carl Zeiss AG, Oberkochen, Germany) at an acceleration voltage of 5–6 kV.

#### 2.3.4. Quasi-Static Tensile Test

An Instron^®^ 5969 electromechanical testing machine (Norwood, MA, USA) was used to perform the uniaxial tensile tests with a load cell of 50 kN. Specimens tested were CM materials, which were cut following the geometry of an ISO 527 type 1BA dumbbell (gauge length of 30 mm; thickness of 1.2 mm). Tensile tests were performed at a crosshead speed of 10 mm/min and the values of yield and fracture properties were the average of at least four replicates. The elastic modulus, ET, of CM and 3D-printed specimens was determined at a crosshead speed of 1 mm/min through an electrical extensometer Instron^®^ 2620–601 (Norwood, MA, USA) with a gauge length of 12.5 mm. The elastic modulus was reported as the average of four specimens and individuated as a secant value between strain levels of 0.05% and 0.25%, according to ISO 527 standard.

#### 2.3.5. Nanoindentation Tests

Hardness and elastic modulus were evaluated with a Berkovich tip by using a nanoindenter machine (Nanomechanics Inc., Oak Ridge, TN, USA) having a declared sensitivity of 3nN for load and 0.1 nm for displacement. The data set was obtained through indentations performed with the mapping test method (Nanoblitz 3d, Nanomechanics, Inc.) of the CM surface, selecting maps of 200 μm × 200 μm square with inside 10 × 10 equidistant indentation points (see Figure S1 in the Supplementary Materials), for a maximum of 45 mN loads. The values of the mechanical properties, an average of 300 measurements, were obtained according to the method proposed by Oliver and Pharr [33,34].

The hardness (H) was evaluated following the formula Equation (4):

(4) $$H=\frac{P}{A}$$

where *P* is the imposed maximum load of 45 mN and *A* is the experimental projected contact area, depending on the geometry of the indenter. The elastic modulus from nanoindentation, *E_n_*, was evaluated from Equation (5) as:

(5) $$En=\frac{1}{2}\frac{dP}{dh}\frac{\sqrt{\pi }}{\sqrt{A}}$$

where *dP*/*dh* is the slope of the unloading section of the elastic–plastic load curve as a function of the indent depth (*h*). We considered 0.33 as a representative value of the Poisson’s coefficient for this hard material as suggested by Fischer-Cripps [35].

#### 2.3.6. Electrical Resistivity Test

Electrical resistivity measurements were carried out under two different configurations. For compounds with low electrical conductivity, the evaluation was provided with the two-probe method. The sample was cut in a square of 50 mm with a thickness of 1.2 mm. Each sample was subjected to a direct current voltage (100 V) by using a Keithley 6517A electrometer/high-resistance meter (Beaverton, OR, USA) and an 8009 resistivity test fixture at room temperature. Compounds with moderately electrical conductivity, which were in the form of filaments and 3D-printed samples (cross-section 6 mm × 2 mm) with a length of 25 mm, were tested at different voltages (2, 5, 12, 24, 30 V) by using a power supply IPS303DD (ISO-TECH, Milan, Italy), according to the ASTM D4496-04 standard under a four-point contact configuration. The current flow between external electrodes was recorded through the IDM 67 Pocket Multimeter electrometer (ISO-TECH, Milan, Italy). The resistivity values represent the average of at least three specimens. The electrical volume resistivity, *ρ*, was evaluated according to the following formula:

(6) $$\rho =R\cdot \frac{S}{L}$$

where *R* is the electrical resistance, *S* is the cross-section area of the specimen, and *L* is the distance between the internal electrodes (L = 3.69 mm).

#### 2.3.7. Thermogravimetric Analysis (TGA)

Thermal degradation properties of 3D samples were investigated through a Q5000 IR thermogravimetric analyzer (TA Instruments-Waters LLC, New Castle, DE, USA) with a sensitivity of <0.1 microgram and weighing accuracy of +/− 0.1%. Specimens of about 15–20 g were tested from 30 °C to 700 °C at a heating rate of 10 °C/min, under a nitrogen flow of 15 mL/min. The temperature of the maximum degradation rate and corresponding peak temperature was determined by DTGA peak (derivative curve of TGA). Residual mass at 700 °C was also referred to as the initial filler content.

#### 2.3.8. Vicat Softening Temperature (VST)

Vicat values were calculated by the HDT-VICAT tester (ATS-Faar S.p.A., Milano, Italy), according to the ASTM D1525-09 standard with a load of 10 ± 0.2 N. Temperature variation ranged from 40 °C to 150 °C with a heating rate of 120 ± 10 °C/hour. Square plates of 10 ± 0.2 mm × 10 ± 0.2 mm × 3.8 ± 0.2 mm were cut from 3D parallelepiped specimens and tested with a distance of the tip at least 3 mm from the edge. VST is defined as the temperature at which the penetration of the circular indenter with a cross-section of 1 mm^2^ reached 1.00 mm. The results represent the average of three specimens.

#### 2.3.9. Heat Deflection Temperature (HDT)

HDT tests were performed by an HDT-VICAT tester (ATS-Faar S.p.A., Milano, Italy), according to the standard ISO 75-2 with an applied stress of 1.80 MPa (method A). Temperature variation ranged from 40 °C to 150 °C with a heating rate of 120 ± 10 °C/h. The required size of the 3D specimens was 80 ± 2 mm × 10 ± 0.2 mm × 3.8 ± 0.2 mm. HDT is defined as the temperature at which a deflection of 0.25 mm is achieved in a three-point bending configuration. The results represent the average of three specimens.

## 3. Results and Discussion

### 3.1. Compression Molding

#### 3.1.1. Melt Flow Index

**Figure 3:**
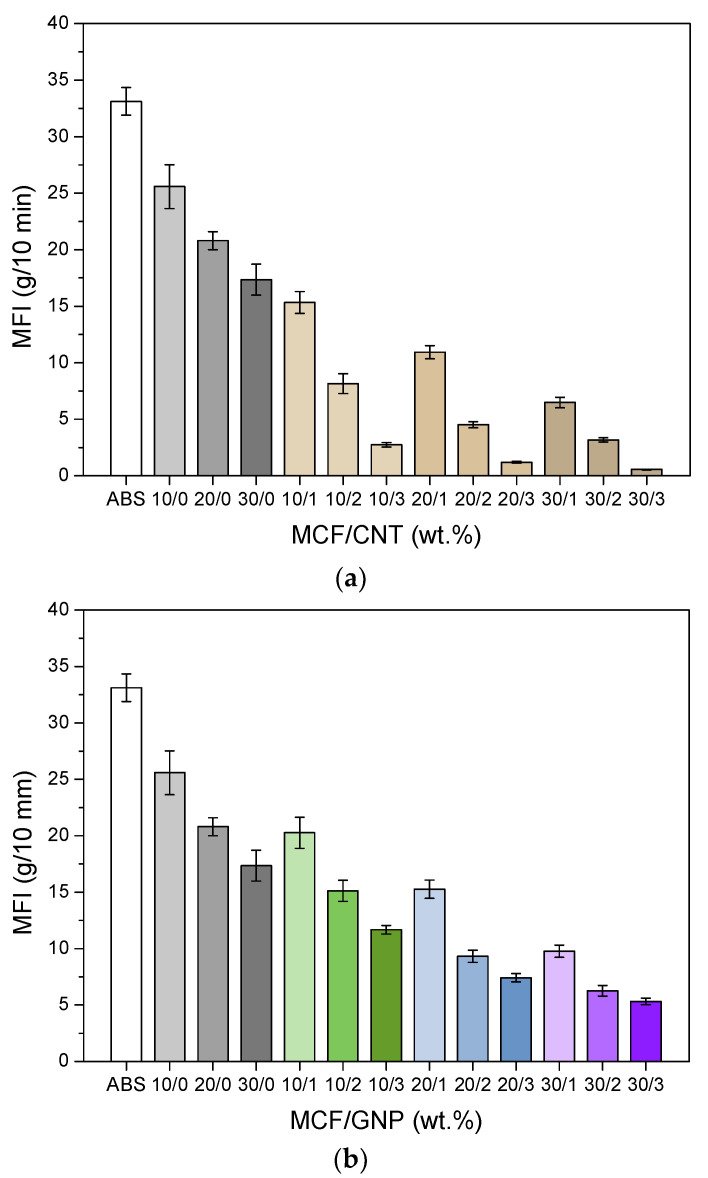
Melt flow index values (220 °C, 10 kg) of neat ABS and multiscale composites as a function of different (**a**) carbon nanotube and (**b**) graphene contents in MCF/CNT and MCF/GNP composites, respectively. The relative ratio of MCF vs. CNT or GNP is reported in percentage by wt. The average values are reported in Table S2 of the Supplementary Materials.

The processability of microfiber composites and multiscale composites was investigated by comparing the melt flow index of ground CM samples. The results pertaining to melt flow index tests are shown in Figure 3.

As expected, the higher the filler content, the lower the melt flow. A relatively slight reduction of MFI was observed after the addition of MCFs in the range of 10–30% by wt., whereas the addition of nanofiller induced a much higher MFI decrease, as expected. In particular, a significant progressive drop in MFI values, 2–3 times lower, was observed in multiscale composites with 1, 2, and 3 wt.% of CNTs. These findings appeared very relevant because of the selection and preparation of multiscale ABS/MCF composites, due to the significant incidence of CNT quantity higher than 1 wt.% [24]. At the same time, it is also important to point out that the effect of GNPs on the MFI of multiscale composites is limited and weaker, even after the nanofiller addition of 3–9 wt.%. The trend is the same as that previously observed in a comparative study on ABS nanocomposites filled with graphene or carbon nanotubes [36]. Experimental results show that the mechanical percolation of the CNTs is achieved with nanofiller quantities greater than 1 wt.%, whereas the processability of these multiscale composites with 2–3 wt.% of CNTs seems to be partially compromised especially at a high MCF content (20–30 wt.%), with MFI in the range of 0.5–5 g/10 min (220 °C, 10 kg).

#### 3.1.2. Density and Morphological Analyses

The evaluation of the bulk density and the volume of voids of the compressed molded samples are shown in Table 4 and compared to the theoretical density. As expected, the bulk density of the composites progressively increases with the content of the filler. However, it is important to note that, simultaneously, the volume of voids also progressively increases up to 0.4% for both the ABS/MCF and ABS/MCF/CNT composites, and about 1.0–1.2% for the ABS/MCF/GNP composites. It appears to be the case that the presence of microvoids (lower than 1.3%) is not only related to the compression molding process under pressure as high as 3.9 MPa, but also depends on the specific shape factor of the filler: 158 for CNTs and about 729 for GNPs. The higher the shape factor, and the higher the filler content, the higher the voids content. For this purpose, the composite aspect ratio (CAR) for each composition has been calculated according to the Equation (7):

CAR = *v*_MCF_ *AR*_MCF_ + *v*_CNT_
*AR*_CNT_ + *v*_GNP_ *AR*_GNP_ (7)

where *v_i_* and *AR_i_* are the volume fraction (see Table S1 in the Supplementary Materials) and the aspect ratio of the i-filler, such as MCF, CNT and GNP respectively.

**Figure 4:**
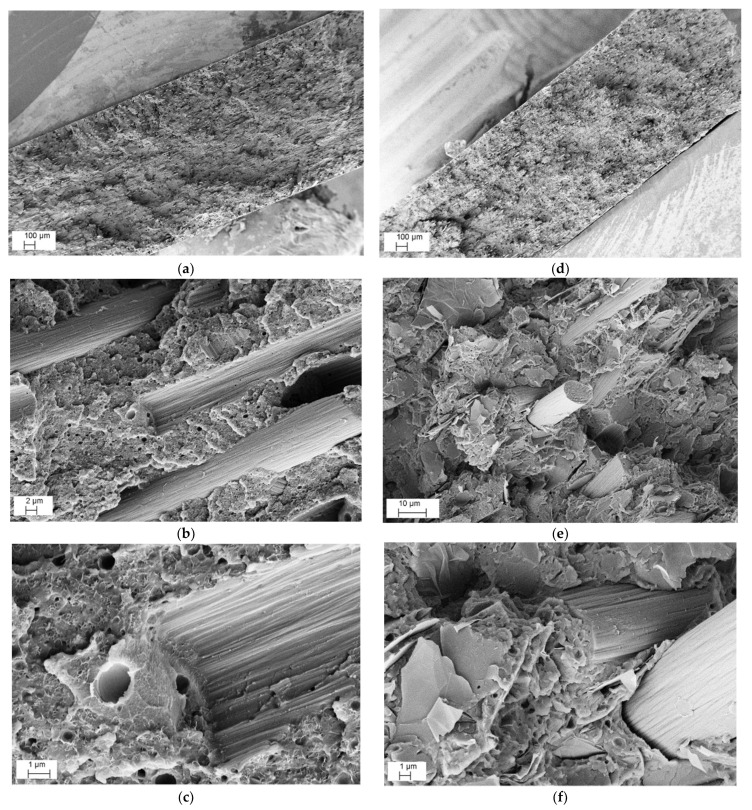
Representative FESEM micrographs at various magnifications of fracture surface of CM_MCF20-CNT3 (**a**–**c**) and CM_MCF20-GNP6 composite (**d**–**f**) plates produced by compression molding.

A direct relation between the CAR and voids content is shown in Table 4. The high pressure of compression molding resulted in minimizing the residual voids of the MCF and CNT composites, due to their relatively low CAR (in the range of 2–10). On the other hand, the much higher CARs ranging from 20 to 75 and residual void rates in the range of 0.75–1.25 percent were determined for the GNP nanocomposites. The SEM micrographs of the fracture surfaces of the ABS/MCF/CNT and ABS/MCF/GNP composite samples at 20 wt.% of MCFs are represented in Figure 4a–f, respectively.

A relatively poor adhesion level between MCFs and ABS is well documented. Pull-out cavities and fiber detachment with contact surfaces of both the matrix and MCFs are evidenced in Figure 4b,e, and a tiny gap around the MCFs is also shown in Figure 4f. Concerning nanofillers, the dispersion of CNTs in the ABS-MCF20-CNT3 sample appeared to be quite good, and no aggregates were observed from SEM microscopy, as shown in Figure 4c, whereas only a relatively good dispersion of GNPs between MCFs was evidenced in the MCF/GNP multiscale composites. In particular, Figure 4f shows the ABS-MCF20-GNP6 sample with a high GNP concentration where graphene flakes appear to be distributed quite evenly within the ABS matrix.

#### 3.1.3. Mechanical Properties

**Figure 5:**
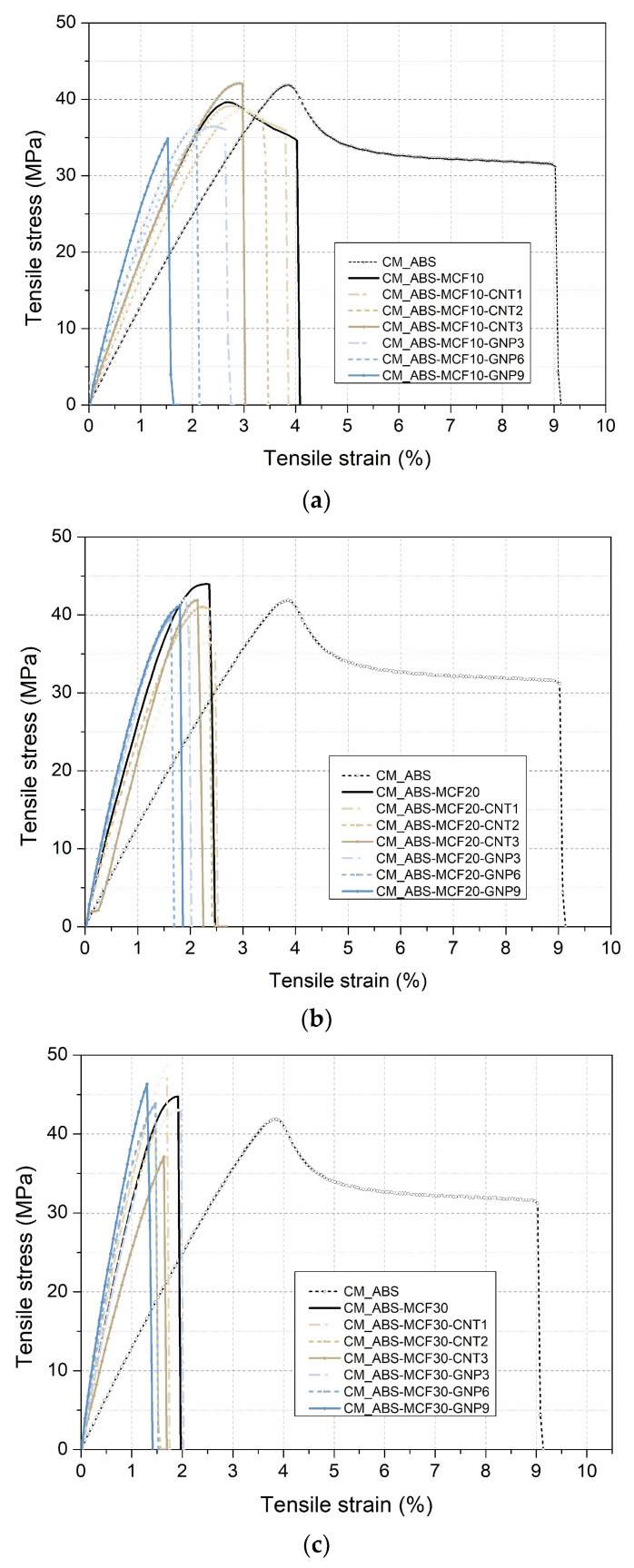
Representative stress–strain curves of multiscale composite CM samples as a function of (**a**) 10 wt.% MCF and different CNT and GNP contents; (**b**) 20 wt.% MCF and different CNT and GNP contents; and (**c**) 30 wt.% MCF and different CNT and GNP contents.

Tensile tests were performed to assess the reinforcing effect of MCFs together with CNTs or GNPs in the ABS composites. Representative stress–strain curves of the ABS multiscale composite of compression-molded samples are reported in Figure 5 and the main results are summarized in Table 5.

As is frequently observed for nanocomposites [37], pure ABS presents a higher strain at break in comparison with the filled samples [36,38,39], and multiscale filler introduction induces a slight further remarkable embrittlement of the samples. If the stiffness and strength of micro composites are considered, a progressive increase of both modulus (E_T_) and stress at break (σ_b_) can be observed, especially up to 20 wt.% of MCFs. Conversely, the maximum stress of composites with MCFs is similar to neat ABS, probably due to the weak adhesion between carbon fibers and the ABS matrix, as already verified on similar composites [7,13,36]. On the other hand, it is interesting to observe the different effect of CNTs or GNPs in multiscale composites This is particularly the case for the presence of microfibers, which increases the modulus (E) to a larger extent than in composites with either MCF and CNT. In fact, the addition of 1–3 wt.% of CNTs negatively affected the stiffening of these multiscale composites with 20 or 30 wt.% of MCFs, probably playing the role of some sort of defect.

On the other side, the graphene filler greatly improves the stiffness values in relation to specimens with the same filler at the microscale, which is consistent with previous studies [38,40]. The presence of graphene could further enhance the stress at break of multiscale composites compared to pure ABS, suppressing on the other hand the yielding in composites, and hence reducing the material toughness, but it is not decisive compared to multiscale composites with MCFs and CNTs. Multiscale composites are mainly influenced by the percentage of MCF reinforcements, and reach stress at break values similar to those of samples with microfibers and different nanofillers, as already verified in previous comparative studies [36].

#### 3.1.4. Nanoindentation Test

**Figure 6:**
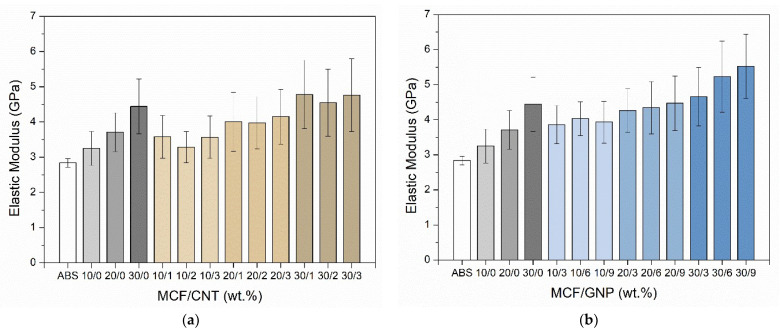
Elastic modulus, En, of CM samples as determined from nanoindentation test at different (**a**) carbon nanotube and (**b**) graphene contents in MCF/CNT and MCF/GNP composites, respectively. The relative ratio of MCF vs. CNT or GNP is reported in percentage by wt.

The elastic modulus (En) and hardness (H) of multiscale composite samples were evaluated by nanoindentation of the CM specimen surface. The higher the filler content, the higher the mechanical properties. The addition of MCFs resulted in an almost linear increase of modulus from 2.8 GPa to about 4.4 GPa. A certain drop of modulus was observed with the addition of CNTs inside the composite with values ranging between 3.3 and 4.8 GPa, as depicted in Figure 6a, whereas almost higher values were measured for GNP multiscale composites in the range of 3.9–5.5 GPa (Figure 6b).

**Figure 7:**
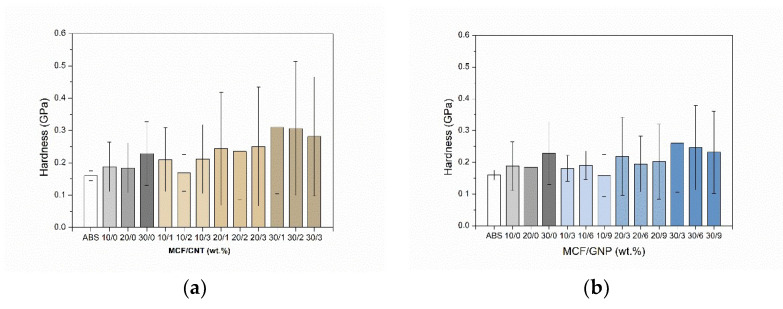
Hardness of CM samples as determined from nanoindentation test at different (**a**) carbon nanotube and (**b**) graphene contents in MCF/CNT and MCF/GNP composites, respectively. The relative ratio of MCF vs. CNT or GNP is reported in percentage by wt.

**Figure 8:**
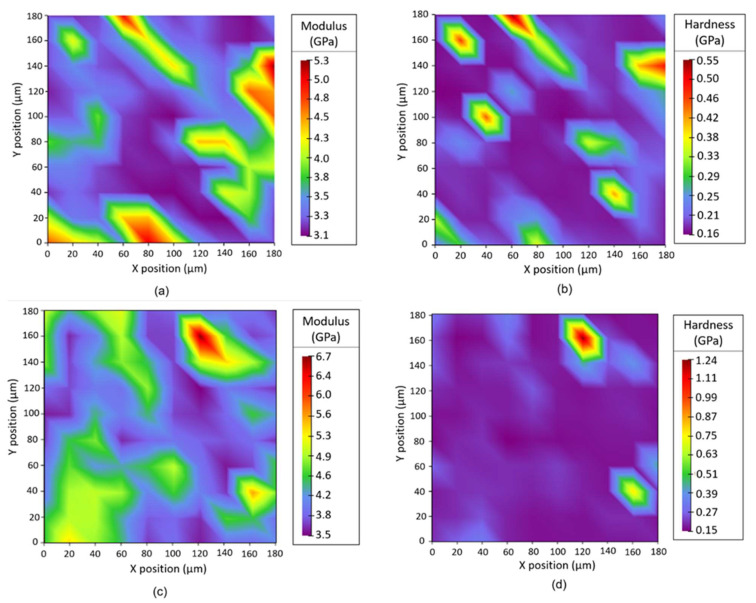
Example of the map of elastic modulus, En (**a**,**c**), and hardness, H (**b**,**d**), of samples CM_MCF10-CNT1 (**a**,**b**) and CM_MCF20-GNP3 (**c**,**d**).

Conversely, the slight difference in the hardness trend between composites with CNT filler (0.18–0.31 GPa) and those with GNP filler (0.16–0.26 GPa) is statistically non-significant, due to the high standard deviation (see Figure 7 and Table S3 in the Supplementary Materials). Through the observation and analysis of the nanoindentation maps and the calculated average values reported in Table S3, it is also possible to define a certain homogeneity in the distribution of the fillers within the composites, as shown in Figure 8. Some filler domains of about 20 μm × 20 μm could be evidenced, from direct measure of the local modulus and harness, as depicted in Figure 8a,b, respectively. For this purpose, it is also worth noting the average size of the imprint of about 10 microns (see Figure S3 in the Supplementary Materials). Moreover, the relative scale of modulus ranges in the interval of 3.1–5.3 GPa for CM_MCF10-CNT1 and 3.5–6.7 GPa for CM_MCF20-GNP3, whereas their average values are 3.5 ± 0.6 GPa and 4.3 ± 0.6 GPa, respectively (See Table S3).

#### 3.1.5. Electrical Resistivity

**Figure 9:**
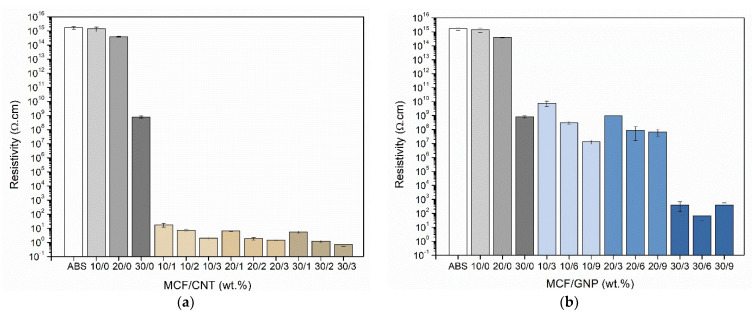
Electrical resistivity of CM samples with different (**a**) carbon nanotube and (**b**) graphene contents in MCF/CNT and MCF/GNP composites, respectively. The relative ratio of MCF vs. CNT or GNP is reported in percentage by wt.

The measurement of electrical resistivity was fundamental for the determination of the electrical percolation threshold and the filler content necessary to obtain an appreciable reduction of electrical resistivity. The results of electricity volume resistivity tests on CM samples are shown in Figure 9.

The addition of CNTs proved to be the most effective way of decreasing the electrical resistivity. Indeed, the reduction in the electrical volume resistivity of the ABS/MCF/GNP composites was about six to seven orders of magnitude lower than that attested for the MCF/ABS bulk samples (between 1.4 × 10^15^ and 8.1 × 10^8^ Ω·cm), as reported in Table S4 in the Supplementary Materials. Furthermore, better results can be achieved with the ABS/MCF/CNT composite samples, for which a reduction of up to fifteen orders of magnitude was registered with respect to pure ABS bulk sample (1.7 × 10^15^ Ω·cm). In particular, a significant drop in resistivity, by more than nine orders of magnitude, can be obtained after the addition of 1 wt.% CNT filler. This percentage is similar to that reported in the literature for ABS/MWCNT nanocomposites for which an electrical percolation threshold of 0.6 wt.% is indicated [40]. The electrical resistivity of samples is measured with the applied voltage between 2 and 100 V. From these findings, ABS/MCF/CNT composites could be considered ohmic conductors. All the details of the various components are shown in Table S4.

### 3.2. Filaments and 3D Printing Samples

Starting from the results of the CM sample tests, the most suitable compositions were selected for filament production and 3D printing. In particular, a preliminary criterion was based on mechanical properties (strength, stiffness, and hardness) and processability (MFI). The higher strength of microfiller composites was found for 20 wt.% of MCFs, in good agreement with Adeniram et al. [17], who showed the better mechanical–compressive properties of ABS/MCF composites produced by additive manufacturing with 20% of the same microfiller used in this study (Panex 30). In order to compare the compositions and to quantify a cumulative effect, a first parameter P_E,MFI_ that maximizes both the stiffness and the processability can be determined from Equation (8):

P_E,MFI_ = E_T_ × MFI (8)

where ET and MFI represent the tensile modulus, and the melt flow index (see Table S2 in the Supplementary Materials). According to this parameter reported in Table 5, the best MCF composition was confirmed and selected as ABS-MCF20. Furthermore, as regards the CNT composites, the three compositions at 1 wt.% of CNT nanofiller were also selected because they exhibited the best combination of processability and stiffness, with P_E,MFI_ in the range of 40–57 GPa g/10 min. Moreover, the composition ABS-MCF20-CNT3 was selected as a good compromise between low resistivity (1.5 Ω·cm) and adequate melt flow (2.7 g/10 min at 220 °C, 10 Kg).

Concerning the second type of nanofiller, GNPs, in multiscale composites at 20 or 30 wt.% of MCFs, the best performing composites in terms of stiffness and melt flow resulted from ABS-MCF20-GNP3, ABS-MCF20-GNP6, and ABS-MCF30-GNP3, with P_E,MFI_ values of 93, 57, and 71 respectively (see Table 5). Other comparative parameters, derived from PE and MFI, also considering hardness (H) or resistivity, were calculated and reported in Tables S3 and S4 of the Supplementary Materials. The selected nine compositions for filament extrusion and 3D printing are reported in Table 2.

In summary, the filament and the 3D-printed samples were produced for the ABS matrix, composite with 20 wt.% MCFs as microfiller, multiscale composites with 1 wt.% CNTs as nanofiller varying the amount of MCFs (10–30 wt.%), and with 3 wt.% GNPs as nanofiller varying the amount of MCFs (20–30 wt.%), plus ABS-MCF20-CNT3 and ABS-MCF20-GNP6 as comparative compositions.

The extrusion of filaments at 240 °C evidenced a certain corrugation of the surface for the higher content composites, in particular all compositions with GNP (see Figure 1). Moreover, ABS-MCF20-GNP6 and ABS-MCF30-GNP3 could not be properly extruded at 240 °C, due to clogging phenomena. This critical aspect could be only partially attributed to the melt flow of materials (see Table S1), but it seemed mainly dependent on the quality of filler, macro- and nano-type, and their total aspect ratio in the composite. The results of the composite aspect ratio, as reported in Table 4, clearly evidenced the predominant role of GNPs, and the higher average CAR values of ABS-MCF20-GNP6 and ABS-MCF30-GNP3, 47 and 27, respectively. Consequently, 3D printing of these compositions was performed at a higher temperature, 280 °C, as previously done for ABS composites with SWCNTs and MWCNTs [41].

#### 3.2.1. Density and Morphological Analysis

The relative density and the volume percentage of the residual voids of filaments and FFF printed samples are reported in Table 6. Filament extrusion of the selected compositions evidenced the formation of products with a very high voids content, between 7 and 28%, much more than that observed in compression molded samples. The difference is attributed to the die-swelling effect during the cooling step of extruded filaments.

After 3D printing, a slight increase of density, especially for GNP composites, and a residual voids content in the range of 15–18% for 20 wt.% or 30 wt.% of MCFs were observed. In particular, it is worth noting that composite filaments and FFF produced with ABS-MCF10-CNT1 exhibited a relatively high density, about 93–94% of the theoretical values, and the lowest residual voids content (6–7%). On the other hand, the density of the other multiscale composite filaments and FFF products was measured in the range of about 73–90% and 80–85%, respectively, of the theoretical values (see Table 4). The volume of voids increases strongly in the production of the filament, probably caused by a combined effect of composition with a relatively high composite aspect ratio (see CAR in Table 4) during the remelting of the material in extrusion, and the subsequent low pressure during cooling in the formation of the filaments. These void volumes are lower in FFF specimens, as a consequence of the second meeting of the thread that occurs during the 3D-printing process, and the relatively fast cooling. The value of the voids seems to settle in the range of 6–18% for MCF/CNT composites, and about 19% for MCF/GNP composites.

**Figure 10:**
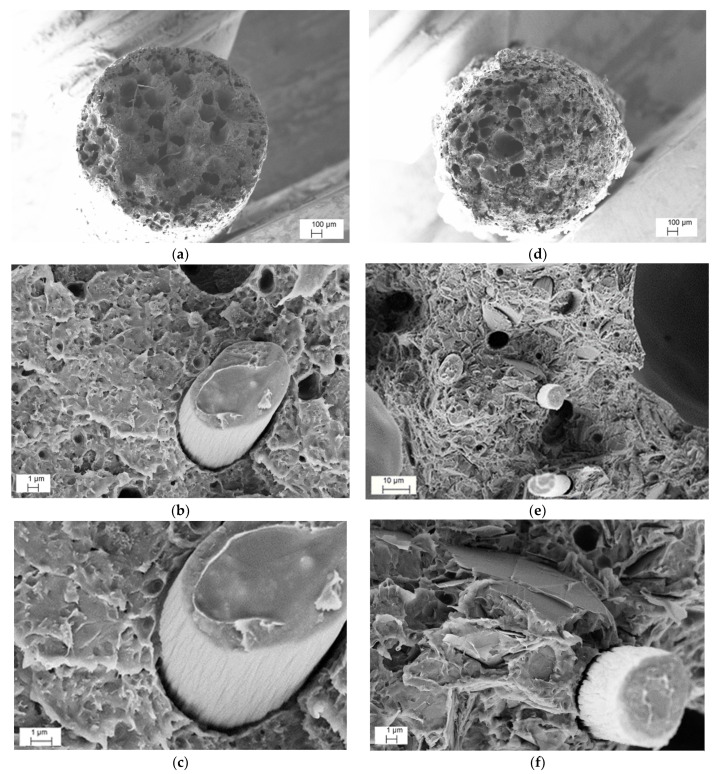
Representative FESEM micrographs at various magnifications of fracture surface of FIL_MCF20-CNT3 (**a**–**c**) and FIL_MCF20-GNP6 (**d**–**f**) composite filaments.

The fracture surface of filaments and 3D-printed specimens was analyzed by SEM. Figure 10 illustrates the SEM images of ABS/MCF/CNT and ABS/MCF/GNP filaments with 20 wt.% of MCFs along with 3 wt.% of CNTs (Figure 10a–c) and 6 wt.% of GNPs (Figure 10d–f) at increasing magnification.

Regarding the CNTs or GNPs addition in both compositions, no aggregates of nanofiller were detected and an almost good local dispersion was observed, indicating a homogenous distribution of single CNT or GNP in the ABS matrix. This suggests that the adopted two-step process, consisting of mixing microfillers and nanofillers in an internal mixer followed by twin-screw extrusion, was capable of avoiding the formation of nanofiller aggregates and properly allowed the dispersion of CNTs or GNPs in the ABS matrix. The presence of voids is also documented. In addition, a fair degree of adhesion level between MCFs and ABS can be observed at a high magnification, as in the compression molded samples shown in Figure 4.

**Figure 11:**
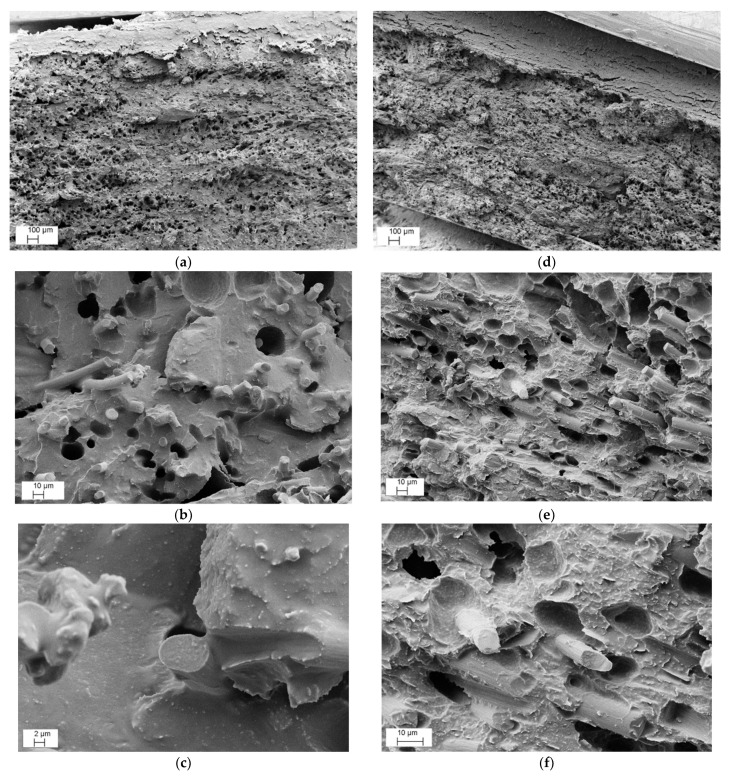
Representative FESEM micrographs at various magnifications of fracture surface of 3D_MCF20-CNT3 (**a**–**c**) and 3D_MCF20-GNP6 (**d**–**f**) composite samples.

Figure 11a–f shows low and high magnifications of the cross-sections of 3D-printed composite specimens loaded with 20% of MCFs and 3% of CNTs (a–c) or 6% of GNPs (d–f), respectively. The presence of voids in 3D-printed specimens is well documented in Figure 11b,e, respectively, as confirmation of the values reported in Table 6 (about 18 and 19 vol%), similarly to filaments. However, it is worth noting the good adhesion between the matrix and MCFs, definitively better than that of the filaments shown in Figure 10. These findings have been attributed to the double steps of processing, where the improved alignment of the carbon fibers could also play a significant role.

#### 3.2.2. Thermal Degradation Behavior

**Figure 12:**
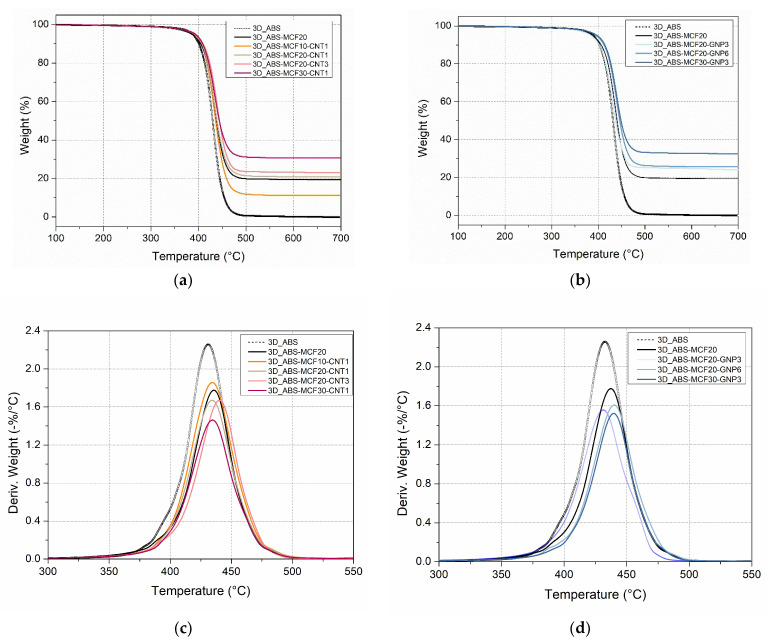
TGA and DTGA curves of multiscale composite 3D-printed samples under nitrogen atmosphere: (**a**,**b**) residual mass as a function of temperature; (**c**,**d**) derivative of the mass loss.

Thermal gravimetric analysis (TGA) was performed to evaluate the thermal stability and composition of the ABS matrix and the prepared composites. Figure 12 shows the TGA thermograms of the pure ABS and composite 3D samples, while the most important parameters are summarized in Table 7. An almost neglectable mass loss at 250 °C (about 0.5–0.6 wt.%) was observed for composites, as an indication of thermal stability of the material in processing conditions. The temperature of DTGA peaks, T_d,max_, shows a tendency to increase after the addition of fillers (Figure 12c–d), and correspondingly the residual mass at the peak is higher.

The maximum mass loss rate (MMLR) is also reduced in composites, where the role of MCF percentage is prevailing; on the other hand, the nanofiller content does not evidence a clear effect on T_d,max_ and MMLR.

The residual mass at 700 °C of the composites proportionally varies according to the composition: for instance, samples with 20 wt.% MCF of filler evidenced a residue of 21% or 23%, as direct dependence on 1 wt.% or 3 wt.% of CNTs or GNPs at the nanoscale level. Either microscale filler (MCFs) or nanoscale filler (CNTs and GNPs) directly contribute to the cumulative residual mass, as a function of their nominal percentage, between 10 and 30 wt.%, or 1 and 3 wt.% and 3 and 9 wt.%, respectively.

#### 3.2.3. Vicat Softening Temperature (VST) and Heat Deflection Temperature (HDT)

The maximum service temperature of 3D-printed materials was evaluated by means of VST and HDT tests, as summarized in Table 8 and documented in Figure S2 (Supplementary Materials). The addition of MCFs evidenced a slight reduction of VST and HDT temperature with respect to pure ABS, attributed to the high voids content (18%). In both tests, the tip penetration or the deflection of the composite samples began at lower temperatures than the corresponding pure ABS bulk sample (see Figure S2) in dependence on the voids content, as evidenced by ABS-MCF10-CNT1. Concerning multiscale composites, results obtained from the Vicat tests showed the tendency of higher VST of CNT composites with respect to GNP composites. In particular, the highest VST temperatures were found for ABS-MCF20-CNT3 and ABS-MCF30-CNT1.

As for the HDT tests, the total filler content in the range of 23 and 33 wt.%. seemed to directly affect the HDT temperature sample composite samples, which were found between 85 and 92 °C (see Table 8).

It is also relevant to observe the effects of voids in HDT composite samples; the higher the voids content, the lower the HDT temperature for both CNT and GNP nanocomposites.

#### 3.2.4. Mechanical Properties

**Figure 13:**
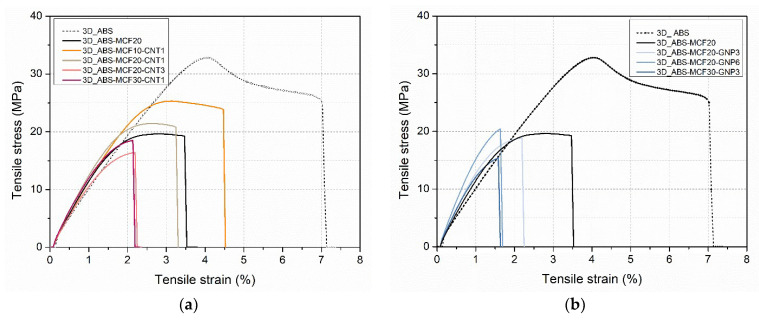
Representative stress–strain curves of multiscale composite 3D-printed samples as a function of different percentages of MCF and CNT (**a**) or GNP (**b**).

Representative stress–strain curves of ABS multiscale composites of 3D-printed samples are reported in Figure 13 and the main results are summarized in Table 9. As previously discussed, neat ABS presents a higher strain at break in comparison with the filled samples [36], and multiscale filler introduction induces further embrittlement of the samples. As for the build configuration, the tensile stress is perpendicular to the cross-section of each layer and the fracture occurs between two printing layers. This configuration implies a low yield strength in the sample.

If an intermediate amount of filler is considered, a certain increase of the elastic modulus (ET) and the progressive disappearance of the yielding can be observed. Stress at yield (σ_y_) of about 19–25 MPa and deformation at the break in the range 2.9–4.3 were measured for multiscale composites up to 21 wt.% of filler. At higher filler content, no yield point and further reduction of strain and stress at break were observed. It is worth noting for ABS that no modulus variation between CM and FFF samples was observed, whereas a reduction of 20% of stress at break was obtained (see σ_b_ ratio = 0.8 in Table 9). Analogously, all 3D-printed composite samples evidenced a significant drop in ductility and strength in comparison with CM sample properties, and the fracture arose at lower strain values than for the pure ABS bulk material. The reduction of strength was in the range of 33–65%, with the σ_b_ ratio between 0.35 and 0.66.

Furthermore, the presence of voids in the specimen structure affects the cross-section during the test, which could be synergistically responsible for the reduction of ductility.

Due to the same printing defects, the elastic modulus in composite 3D samples was also reduced with respect to the CM samples at the same composition. The reduction of stiffness was in the range of 15–57%, with the ET ratio between 0.43 and 0.85. The higher the filler content, the larger the reduction of stiffness and strength, especially in the case of GNP multiscale composites.

#### 3.2.5. Electrical Resistivity Properties

**Figure 14:**
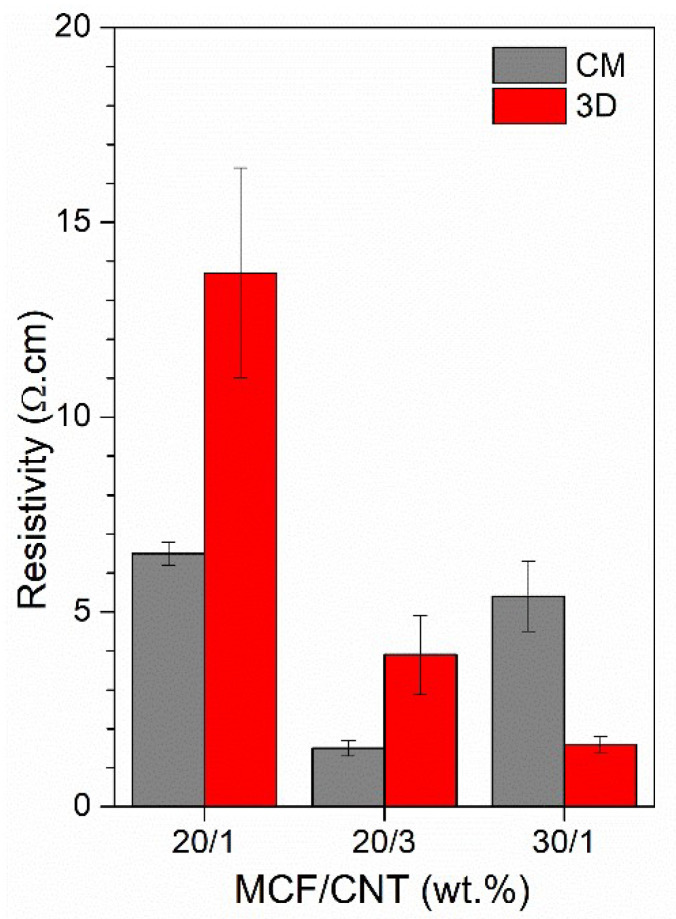
Electrical resistivity of CM and 3D-printed samples in different MCF and CNT contents.

Between the eight 3D-printed samples of ABS multiscale composites, only the formulations with CNTs and MCFs at 20 wt.% or 30 wt.% exhibited a minimum conductivity for measurement. Results about the electrical resistivity of 3D-printed multiscale composites are shown in Figure 14 (details of their values are summarized in Table S5 in the Supplementary Materials) and compared to those of the CM samples. The ABS-MCF20-CNT1 and ABS-MCF20-CNT3 compositions denoted a slight loss of conductivity of 3D-printed samples with respect to compression-molded samples; in fact, a 3D vs. CM resistivity ratio of about 2.3 was determined (see Table S5 in Supplementary), despite the fact that their void volumes had enormously higher values. At the same time, it is also worth noting the low resistivity of 3D_ABS-MCF30-CNT1, 1.6 Ω·cm, which is the lowest of the 3D-printed specimens. This value was reduced to about one-third of that of the corresponding CM samples, probably independent of the double extrusion process of filament extrusion and 3D printing, which promoted a better distribution and network of the conductive fillers. The resistivity data of these 3D samples resulted in the same order of magnitude of ABS nanocomposites containing 10 wt.% of MWCNTs (4.0 ± 1.7 Ω·cm) or SWCNTs (10.7 ± 0.6 Ω·cm) [41], or of the hybrid GNP/CNT 6 wt.% nanocomposite with a relative fraction in the range of 0.7–0.3 (resistivity between 12.7 and 1.9 Ω·cm, respectively) [28].

## 4. Conclusions

The novelty of this research is the appropriate compounding, process, and characterization of multiscale carbonaceous ABS composites based on microfibers (MCFs) and nanofiller (CNTs or GNPs) at different percentage ratios by means of a solvent-free process. As for the effects of fillers, the mechanical properties (modulus and strength) of the compression-molded ABS composite samples were increased by the addition of microfillers (CNTs and GNPs), even though, as expected, the strain at break values was reduced by microfiller addition (MCFs). The electrical conductivity was improved by the nanofillers compared to pure ABS and micro composites and the best performance was reached with the CNT filler.

Multiscale ABS/MCF/GNP composites had a good impact on the mechanical properties of compression-molded samples. Conversely, significant improvements in electrical conductivity were obtained by ABS/CNTs.

As for the production process, it greatly influenced the density of samples and, therefore, their mechanical, electrical, and thermal properties. In particular, 3D-printed samples presented a drastic loss of ductility (in the range of 33–65%) if compared with the CM specimens due to the presence of voids, even though electrical conductivity could be maintained for some compositions. Interestingly, the printing process affected the composites differently, causing overall deterioration in the mechanical performances of the ABS/MCF/GNP material compared to the ABS/MCF/CNT printed composite. As regards the conductive properties of the compression-molded samples, all multiscale composites with CNTs exhibited a low resistivity in the range of 0.7–18 Ω·cm. The best conductive behavior of the FFF product was shown by ABS-MCF30-CNT1, with a resistivity of 1.6 Ω·cm.

Selective and comparative parameters were also presented in order to evaluate the most suitable compositions of these multiscale ABS composites, taking into account their processability, and the resulting mechanical and/or conductive properties because of the proper applications, such as for thermoelectric devices or sensors.

## Figures and Tables

**Table 1:** Properties of carbonaceous fillers used in this study according to the manufacturer.

Filler Code	Filler Type	Manufacturer	Density (g/cm^3^)	Length/Width(μm)	Diameter/ Thickness (nm)	Aspect Ratio	Surface Area (m^2^/g)	Carbon Purity (%)
MCF	PX30	Zoltek, St. Louis, MO, USA	1.75 ^a^	100–150	7200	14–21	-	>99
CNT	MWCNT-NC7000 ^b^	Nanocyl, Belgium	2.15 ± 0.03 ^b^	1.5	9.5	158	250–300	>90
GNP	xGnP-M5 ^c^	XG Sciences, Lansing, MI, USA	2.06 ± 0.03 ^c^	5	6–8	625–833	120–150	>99.5

^a^ From Reference [30]; ^b^ from Reference [31]; ^c^ from Reference [32].

**Table 2:** Designation and formulation of ABS multiscale composites at different MCF/CNT and MCF/GNP percentages and fillers’ ratios, and processing techniques: compression molding (CM), filament extrusion (Filament), and 3D printing.

Sample	Material Composition					Processing Technique		
	ABS (wt.%)	MCF (wt.%)	CNT (wt.%)	GNP (wt.%)	Nanofiller/MCF Ratio	CM	Filament	3D Printing
**ABS**	100	-	-	-	-	X	X	X
**ABS-MCF10**	90	10	-	-	0	X	-	-
**ABS-MCF20**	80	20	-	-	0	X	X	X
**ABS-MCF30**	70	30	-	-	0	X	-	-
**ABS-MCF10-CNT1**	89	10	1	-	0.1	X	X	X
**ABS-MCF10-CNT2**	88	10	2	-	0.2	X	-	-
**ABS-MCF10-CNT3**	87	10	3	-	0.3	X	-	-
**ABS-MCF20-CNT1**	79	20	1	-	0.05	X	X	X
**ABS-MCF20-CNT2**	78	20	2	-	0.1	X	-	-
**ABS-MCF20-CNT3**	77	20	3	-	0.15	X	X	X
**ABS-MCF30-CNT1**	69	30	1	-	0.03	X	X	X
**ABS-MCF30-CNT2**	68	30	2	-	0.07	X	-	-
**ABS-MCF30-CNT3**	67	30	3	-	0.1	X	-	-
**ABS-MCF10-GNP3**	87	10	-	3	0.3	X	-	-
**ABS-MCF10-GNP6**	84	10	-	6	0.6	X	-	-
**ABS-MCF10-GNP9**	81	10	-	9	0.9	X	-	-
**ABS-MCF20-GNP3**	77	20	-	3	0.15	X	X	X
**ABS-MCF20-GNP6**	74	20	-	6	0.3	X	X	X
**ABS-MCF20-GNP9**	71	20	-	9	0.45	X	-	-
**ABS-MCF30-GNP3**	67	30	-	3	0.1	X	X	X
**ABS-MCF30-GNP6**	64	30	-	6	0.2	X	-	-
**ABS-MCF30-GNP9**	61	30	-	9	0.3	X	-	-

**Table 3:** Dimensions and processing parameters of FFF specimens.

Sample	X (mm)	Y (mm)	Z (mm)	Deposition Time of a Single Layer (s)	Number of Layers	Total Time (min)	Testing
Dumbbell	75.0	5–10	2.0	123	10	20.5	Density, tensile test, resistivity
Parallelepiped	80.0	10.0	3.8	123	19	39.0	TGA, VST, and HDT

**Table 4:** Theoretical density (*ρ_c_*), experimental density (*ρ_m_*), and volume of void content (*V_v_*) of compression-molded (CM) samples. Composite aspect ratio (CAR) is also reported, as calculated according to Equation (7).

Sample	*ρ_c_* (g/cm^3^)	*ρ_m_* (g/cm^3^)	*V_v_* (%)	CAR
CM_ABS	1.040	1.040 ± 0.000	0.00	//
CM_ABS-MCF10	1.083	1.081 ± 0.000	0.16	1.1
CM_ABS-MCF20	1.130	1.130 ± 0.002	0.01	2.3
CM_ABS-MCF30	1.180	1.177 ± 0.001	0.30	3.6
CM_ABS-MCF10-CNT1	1.089	1.086 ± 0.001	0.30	2.7
CM_ABS-MCF10-CNT2	1.095	1.092 ± 0.000	0.31	3.9
CM_ABS-MCF10-CNT3	1.101	1.098 ± 0.001	0.26	5.1
CM_ABS-MCF20-CNT1	1.136	1.133 ± 0.001	0.24	5.1
CM_ABS-MCF20-CNT2	1.143	1.140 ± 0.001	0.25	6.7
CM_ABS-MCF20-CNT3	1.149	1.147 ± 0.000	0.18	8.2
CM_ABS-MCF30-CNT1	1.187	1.183 ± 0.001	0.37	6.8
CM_ABS-MCF30-CNT2	1.194	1.193 ± 0.001	0.14	8.4
CM_ABS-MCF30-CNT3	1.202	1.200 ± 0.002	0.15	10.0
CM_ABS-MCF10-GNP3	1.100	1.089 ± 0.001	1.00	23.6
CM_ABS-MCF10-GNP6	1.118	1.105 ± 0.001	1.10	45.5
CM_ABS-MCF10-GNP9	1.136	1.124 ± 0.001	1.04	67.4
CM_ABS-MCF20-GNP3	1.148	1.138 ± 0.001	0.88	25.4
CM_ABS-MCF20-GNP6	1.167	1.158 ± 0.001	0.77	47.2
CM_ABS-MCF20-GNP9	1.187	1.172 ± 0.002	1.27	69.1
CM_ABS-MCF30-GNP3	1.201	1.191 ± 0.000	0.77	27.1
CM_ABS-MCF30-GNP6	1.222	1.210 ± 0.001	0.98	49.0
CM_ABS-MCF30-GNP9	1.243	1.228 ± 0.001	1.24	70.9

**Table 5:** Tensile modulus (ET), yield stress (σ_y_), stress at break (σ_b_), and deformation at break (ε_b_) of quasi-static tensile properties of ABS multiscale CM samples. Selection parameters P_E,MFI_ are based on product ET and MFI, according to Equation (8) (the bold values evidence the selected compositions for FFF).

Sample	E_T_ (MPa)	σ_y_ (MPa)	σ_b_ (MPa)	ε_b_ (%)	P_E,MFI_ ×10^−3^ * (GPa g/10 min)
CM_ABS	2313 ± 38	41.4 ± 0.8	31.2 ± 0.5	10.1 ± 5.6	**76.6**
CM_ABS-MCF10	3752 ± 324	39.8 ± 0.6	34.8 ± 1.2	4.2 ± 0.5	96.1
CM_ABS-MCF20	6000 ± 499	n.d. **	44.2 ± 1.5	2.1 ± 0.3	**124.8**
CM_ABS-MCF30	6586 ± 1614	n.d. **	42.9 ± 3.0	1.8 ± 0.1	114.6
CM_ABS-MCF10-CNT1	3609 ± 455	38.8 ± 1.2	36.5 ± 0.9	3.7 ± 1.1	**55.2**
CM_ABS-MCF10-CNT2	3858 ± 396	39.0 ± 0.6	36.3 ± 2.2	3.5 ± 0.2	31.3
CM_ABS-MCF10-CNT3	3912 ± 508	n.d. **	42.2 ± 1.0	2.9 ± 0.2	10.6
CM_ABS-MCF20-CNT1	5165 ± 621	n.d. **	38.7 ± 3.3	2.2 ± 0.5	**56.3**
CM_ABS-MCF20-CNT2	5305 ± 1077	n.d. **	41.6 ± 2.8	2.3 ± 0.2	23.8
CM_ABS-MCF20-CNT3	5336 ± 848	n.d. **	41.7 ± 1.6	2.0 ± 0.2	**6.4**
CM_ABS-MCF30-CNT1	6388 ± 1369	n.d. **	46.2 ± 5.2	1.9 ± 0.1	**41.5**
CM_ABS-MCF30-CNT2	6367 ± 533	n.d. **	41.3 ± 1.7	1.6 ± 0.1	20.4
CM_ABS-MCF30-CNT3	5739 ± 560	n.d. **	36.5 ± 1.7	1.6 ± 0.2	2.9
CM_ABS-MCF10-GNP3	3658 ± 212	36.3 ± 1.3	34.3 ± 3.1	2.8 ± 0.1	74.3
CM_ABS-MCF10-GNP6	4025 ± 313	n.d. **	33.2 ± 4.7	2.2 ± 0.2	60.8
CM_ABS-MCF10-GNP9	4760 ± 560	n.d. **	35.7 ± 2.3	1.7 ± 0.2	55.7
CM_ABS-MCF20-GNP3	6101 ± 187	n.d. **	42.6 ± 1.8	2.0 ± 0.2	**93.3**
CM_ABS-MCF20-GNP6	6104 ± 127	n.d. **	39.8 ± 2.0	1.7 ± 0.1	**56.8**
CM_ABS-MCF20-GNP9	6406 ± 675	n.d. **	41.8 ± 2.0	1.7 ± 0.1	47.4
CM_ABS-MCF30-GNP3	7208 ± 1390	n.d. **	42.8 ± 2.9	1.8 ± 0.1	**70.6**
CM_ABS-MCF30-GNP6	8239 ± 937	n.d. **	45.1 ± 2.7	1.5 ± 0.1	51.9
CM_ABS-MCF30-GNP9	9193 ± 1030	n.d. **	47.7 ± 3.0	1.4 ± 0.1	48.7

* See Equation (8). n.d. **: not detectable (see Figure 5).

**Table 6:** The relative density and the volume of the void of filaments and 3D-printed samples.

Sample	Filament		3D-Printed Sample	
	*ρ_c_* (g/cm^3^)	*V_v_* (%)	*ρ_c_* (g/cm^3^)	*V_v_* (%)
ABS	1.045 ± 0.002	0.0	1.044 ± 0.002	0.0
ABS-MCF20	0.919 ± 0.025	18.9	0.930 ± 0.006	17.9
ABS-MCF10-CNT1	1.015 ± 0.003	7.2	1.024 ± 0.005	6.2
ABS-MCF20-CNT1	1.021 ± 0.025	10.5	0.968 ± 0.003	15.0
ABS-MCF20-CNT3	0.851 ± 0.008	26.2	0.937 ± 0.013	18.0
ABS-MCF30-CNT1	1.001 ± 0.041	15.9	0.992 ± 0.038	16.7
ABS-MCF20-GNP3	0.883 ± 0.015	23.4	0.938 ± 0.014	19.9
ABS-MCF20-GNP6	0.875 ± 0.023	25.3	0.938 ± 0.012	19.1
ABS-MCF30-GNP3	0.873 ± 0.003	27.5	0.973 ± 0.007	18.5

**Table 7:** TGA data of pure ABS and multiscale composite printed samples in a nitrogen atmosphere.

Sample	Residual Mass at 250 °C (wt.%)	T_d,max_ ^1^ (°C)	Residual Mass at Peak (wt.%)	MMLR (%/°C)	Residual Mass at 700 °C (wt.%)
3D_ABS	99.3	431.1	47.6	−2.26	0.0
3D_ABS-MCF20	99.4	434.6	55.2	−1.78	19.4
3D_ABS-MCF10-CNT1	99.4	433.3	53.4	−1.86	11.2
3D_ABS-MCF20-CNT1	99.4	432.5	58.0	−1.67	20.9
3D_ABS-MCF20-CNT3	99.4	437.8	56.6	−1.67	22.9
3D_ABS-MCF30-CNT1	99.4	433.4	62.8	−1.46	30.7
3D_ABS-MCF20-GNP3	99.5	429.8	59.2	−1.56	23.2
3D_ABS-MCF20-GNP6	99.4	437.4	59.6	−1.61	25.5
3D_ABS-MCF30-GNP3	99.5	437.9	63.0	−1.52	32.1

T_d,max_^1^ = maximum degradation rate temperature, MMLR = maximum mass loss rate.

**Table 8:** Vicat softening temperature (VST) and Heat deflection temperature (HDT) of 3D-printed pure ABS and selected multiscale composites.

Sample	VST (10 N) (°C)	HDT (1.8 MPa) (°C)
3D_ABS	107.2 ± 1.4	90.3 ± 3.6
3D_ABS-MCF20	104.5 ± 1.0	88.7 ± 4.0
3D_ABS-MCF10-CNT1	106.3 ± 1.2	92.0 ± 3.6
3D_ABS-MCF20-CNT1	107.0 ± 0.7	90.3 ± 4.5
3D_ABS-MCF20-CNT3	111.0 ± 1.2	87.0 ± 0.2
3D_ABS-MCF30-CNT1	110.0 ± 1.4	91.0 ± 2.7
3D_ABS-MCF20-GNP3	105.3 ± 1.1	85.3 ± 1.0
3D_ABS-MCF20-GNP6	107.5 ± 1.4	87.3 ± 1.7
3D_ABS-MCF30-GNP3	108.8 ± 1.1	91.7 ± 0.8

**Table 9:** Tensile modulus (E_T_), yield stress (σ_y_), stress at break (σ_b_), and deformation at break (ε_b_) of quasi-static tensile properties of ABS multiscale 3D-printed samples.

Sample	E_T_ (MPa)	σ_y_ (MPa)	σ_b_ (MPa)	ε_b_ (%)	E ratio 3D/CM	σ_b_ ratio 3D/CM
3D_ABS	2345 ± 149	32.3 ± 0.5	24.9 ± 2.2	8.2 ± 2.9	1.01	0.80
3D_ABS-MCF20	3131 ± 280	19.7 ± 0.5	19.4 ± 0.6	3.4 ± 0.2	0.52	0.44
3D_ABS-MCF10-CNT1	3072 ± 77	25.5 ± 0.6	24.2 ± 0.7	4.3 ± 0.3	0.85	0.66
3D_ABS-MCF20-CNT1	3353 ± 193	21.1 ± 0.9	20.4 ± 1.1	2.9 ± 0.2	0.65	0.53
3D_ABS-MCF20-CNT3	4019 ± 340	n.d. *	18.4 ± 5.2	1.8 ± 0.3	0.75	0.44
3D_ABS-MCF30-CNT1	2882 ± 355	n.d. *	17.4 ± 1.6	2.1 ± 0.1	0.45	0.38
3D_ABS-MCF20-GNP3	3053 ± 389	n.d. *	19.7 ± 1.9	1.8 ± 0.1	0.50	0.46
3D_ABS-MCF20-GNP6	3509 ± 515	n.d. *	20.5 ± 1.8	1.5 ± 0.1	0.57	0.52
3D_ABS-MCF30-GNP3	3091 ± 707	n.d. *	14.9 ± 3.1	1.3 ± 0.1	0.43	0.35

n.d. *: not detectable (see Figure 13).

## Data Availability

Not applicable.

## Supplementary Materials

The following supporting information can be downloaded at: https://www.mdpi.com/article/10.3390/nano12122064/s1, Figure S1: Example of an optical microscopy of CM nanocomposites tested surface after nanoindentation (view of 8 × 4 indents). The chosen set-up of 10 × 10 imprints in the 200 um × 200 um area is the minimum one that ensures adequate mapping of the properties, Figure S2: Representative 3D dumbbell specimens of ABS-MCF20 composites before (top) and after (bottom) mechanical test, Figure S3: Magnified view of the clamping side of 3D dumbbell specimens before (top) and after (bottom) mechanical test, Figure S4: Effect of nano reinforcement on VST (a,b) and HDT (c,d) thermograms of multiscale composite 3D-printed samples with CNT (left) and GNP (right), Table S1: The volume fraction of ABS multiscale composites at various contents of MCF, CNT, and GNP. The aspect ratio of the single components and their combination in composites, composite aspect ratio, is also reported (calculated according to Equation (7)), Table S2: Average melt flow index (MFI) at 10 kg and 220 °C of ABS multiscale composites (from CM samples), Table S3: Results of nanoindentation tests of ABS multiscale CM samples. The results of elastic modulus (En) and hardness (H) are reported as an average of 300 measurements. Selection parameter P2 = P_E,MFI,H_ is also reported according to Equation (S1). In column P2, the bold values evidence the selected compositions for FFF, Table S4: Electrical resistivity (Res) of ABS multiscale CM samples as a function of the applied voltage. Selection parameter P3 = P_E,MFI,R_ is also reported according to Equation (S2), Table S5: Electrical resistivity (Res) of ABS multiscale 3D-printed samples as a function of the applied voltage. Comparative resistivity ratio between 3D-printed and compression-molded samples.
