# Supplementary material for: Three Dimensional Printing of Multiscale Carbon Fiber-Reinforced Polymer Composites Containing Graphene or Carbon Nanotubes
Source: Nanomaterials (Basel). 2022 Jun 15;12(12):2064. doi: 10.3390/nano12122064 (PMC9227618; doi:10.3390/nano12122064)
Supplement: Supplementary file 1 [file nanomaterials-12-02064-s001.zip › nanomaterials-1743113-supplementary final.pdf]

nanomaterials

Supplementary

Three Dimensional Printing of Multiscale Carbon Fiber-Reinforced Polymer Composites Containing Graphene or Carbon Nanotubes

Sara Residori [1], Sithiprumnea Dul [2], Alessandro Pegoretti [2], Luca Fambri [2,*] and Nicola M. Pugno [1,3,*]

1 Laboratory for Bioinspired, Bionic, Nano, Meta Materials & Mechanics, Department of Civil, Environmental and Mechanical Engineering, University of Trento, Via Mesiano, 77, 38123 Trento, Italy; sara.residori@unitn.it
2 Department of Industrial Engineering and INSTM Research Unit, University of Trento, Via Sommarive, 9, 38123 Trento, Italy; sithiprumnea_dul@yahoo.com (S.D.); alessandro.pegoretti@unitn.it (A.P.)
3 School of Engineering and Material Science, Queen Mary University of London, Mile End Road, London E1 4NS, UK
* Correspondence: luca.fambri@unitn.it (L.F.); nicola.pugno@unitn.it (N.M.P.)

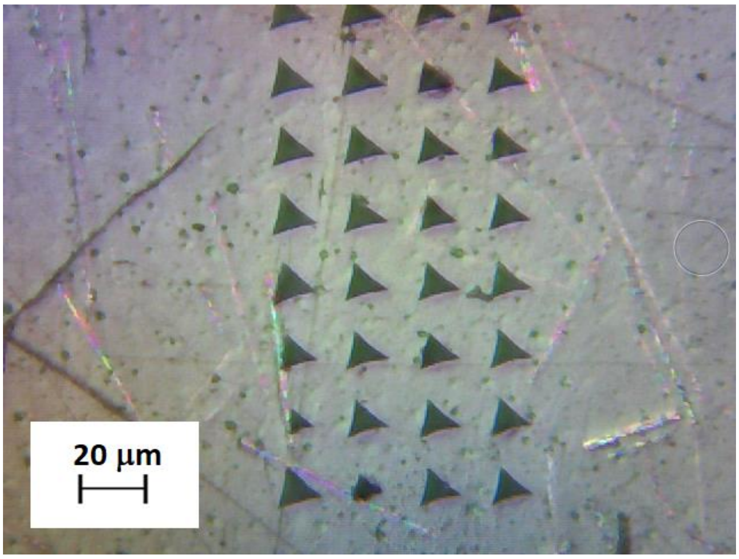

Figure S1. Example of an optical microscopy of CM nanocomposites tested surface after nanoindentation (8 × 4 indents). The chosen set-up of 10 × 10 imprints in the 200 um × 200 um area is the minimum one that ensures adequate mapping of the properties. The chosen set-up of 10 × 10 imprints in the 200 × 200 um area is the minimum one that ensures adequate mapping of the properties.

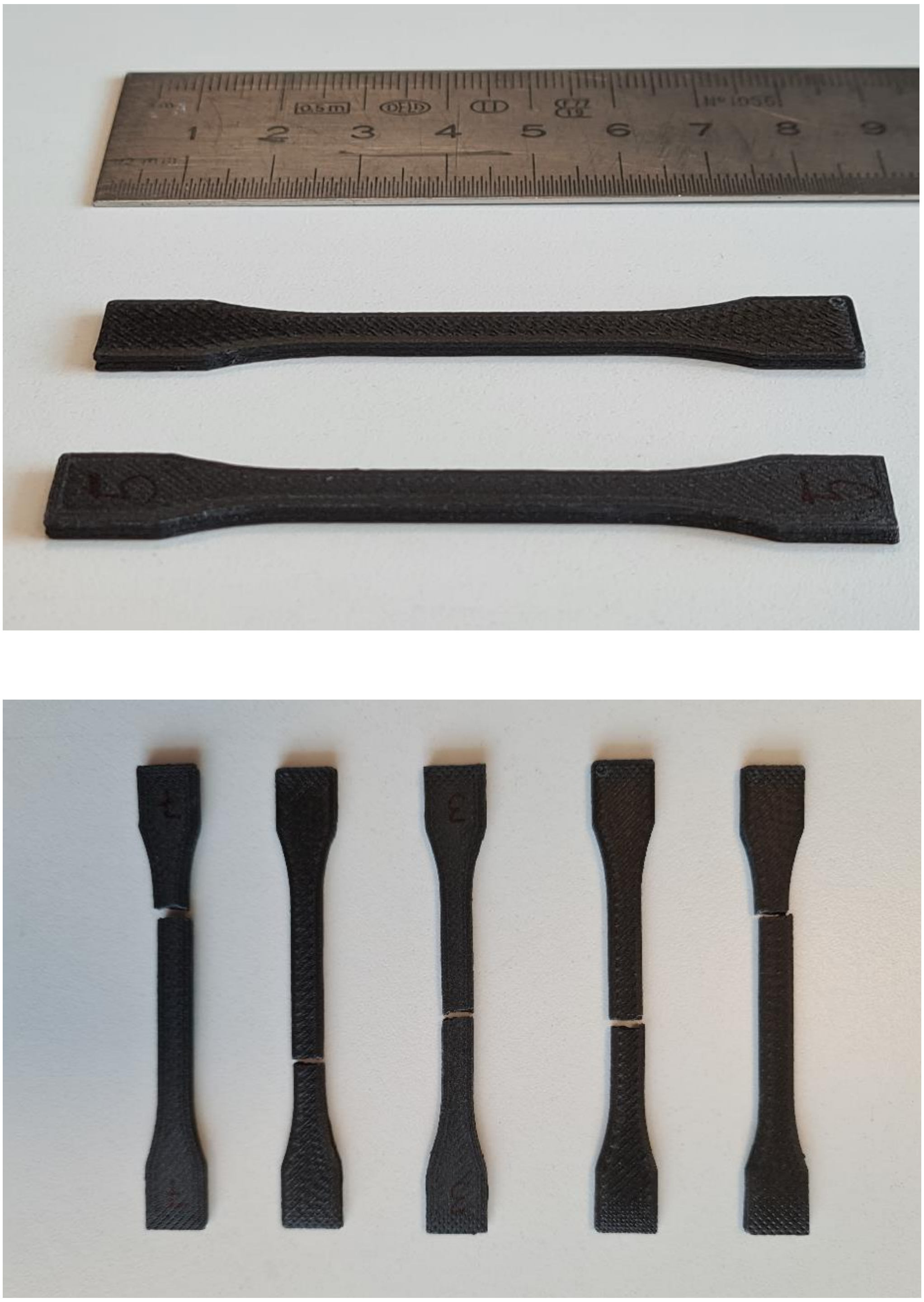

Figure S2. Representative 3D dumbbell specimens of ABS-MCF20 composites before (top)) and after (bottom) mechanical test.

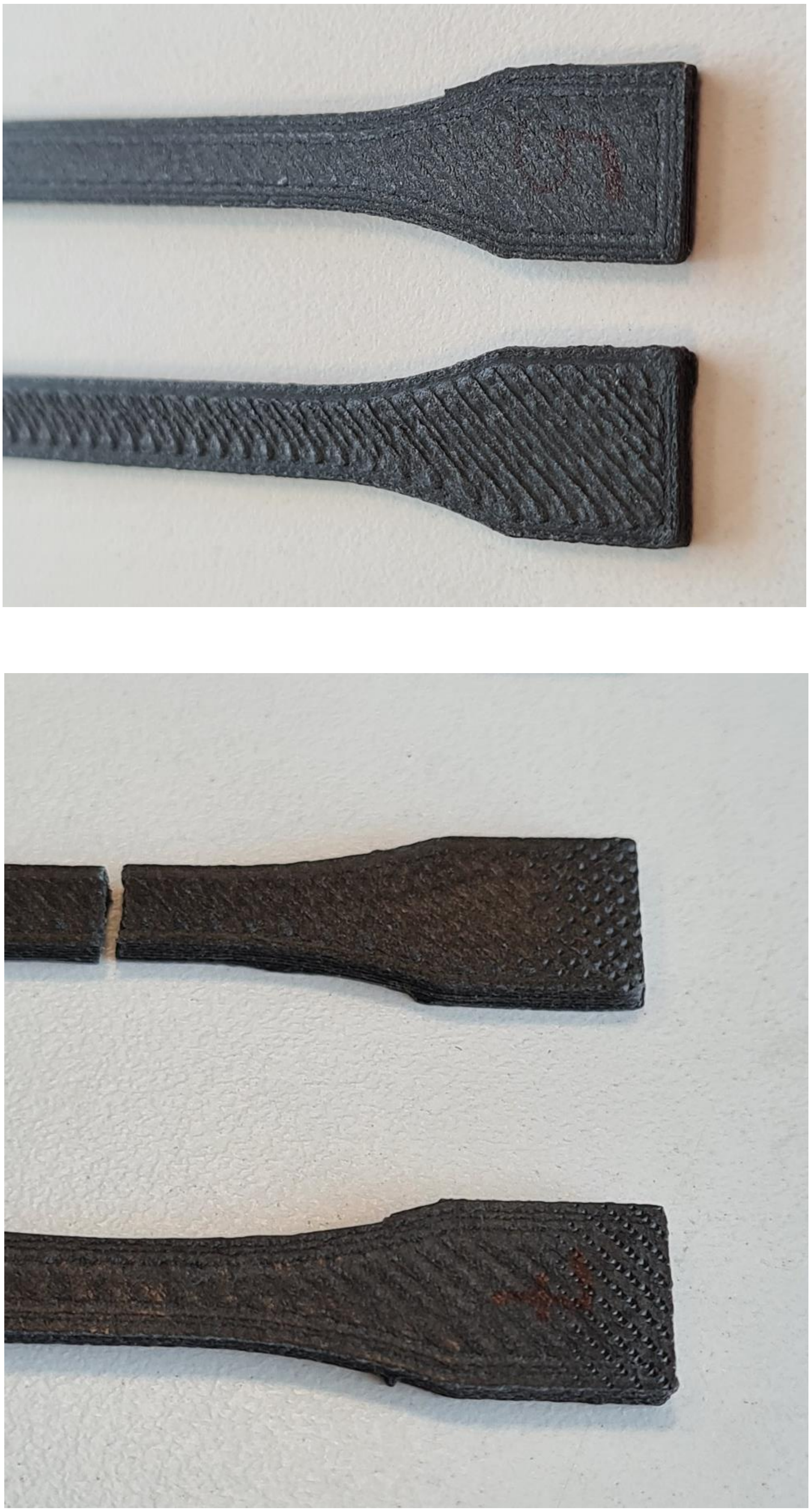

Figure S3. Magnified view of the clamping side of 3D dumbbell specimens before (top) and after (bottom) mechanical test.

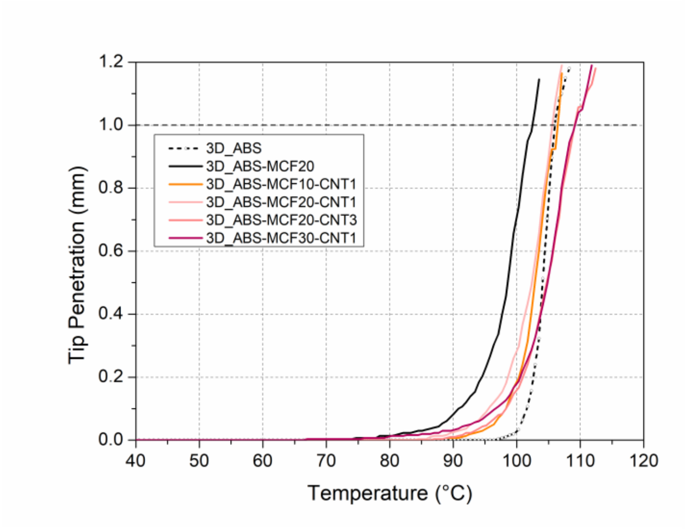

(**a**)

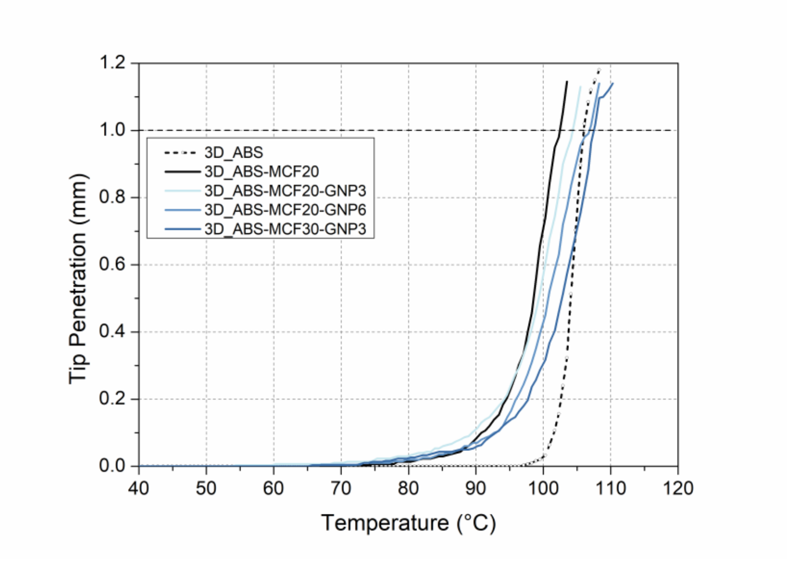

(**b**)

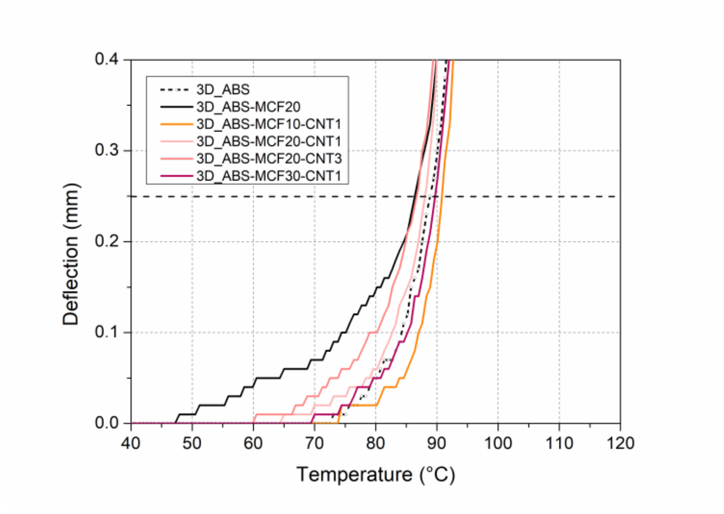

(**c**)

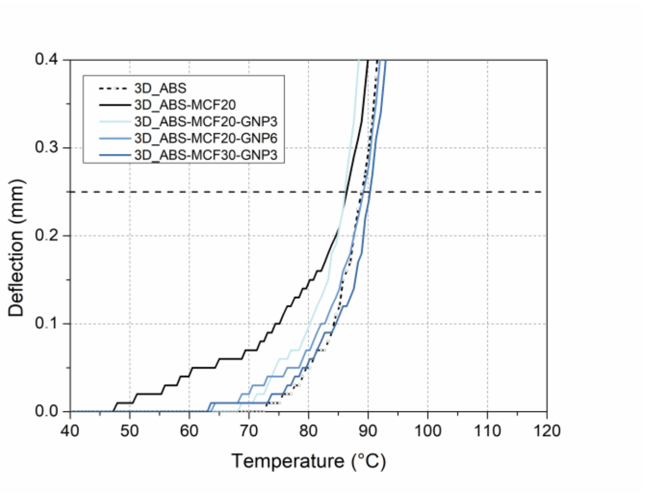

(**d**)

Figure S4. Effect of nano reinforcement on VST (**a**, **b**) and HDT (**c**, **d**) thermograms of multiscale composite 3D-printed samples with CNT (left) and GNP (right).

Table S1. The volume fraction of ABS multiscale composites at various content of MCF, CNT, and GNP. The Aspect Ratio of the single components and their combination in composites, Composite Aspect Ratio, is also reported (calculated according to Equation (7)).

**Sample**	**Material composition**				**Aspect Ratio and CAR**		
	**ABS**	**MCF**	**CNT**	**GNP**	**min**	**max**	**average**
ABS	1.000	0.000	0.000	0.000	//	//	
MCF	0.000	1.000	0.000	0.000	14	21	17.5
CNT	0.000	0.000	1.000	0.000	158	158	158
GNP	0.000	0.000	0.000	1.000	625	833	729
ABS-MCF10	0.938	0.062	0.000	0.000	0.9	1.3	1.1
ABS-MCF20	0.871	0.129	0.000	0.000	1.8	2.7	2.3
ABS-MCF30	0.797	0.203	0.000	0.000	2.8	4.3	3.6
ABS-MCF10-CNT1	0.933	0.062	0.005	0.000	1.7	3.7	2.7
ABS-MCF10-CNT2	0.927	0.063	0.010	0.000	2.5	5.3	3.9
ABS-MCF10-CNT3	0.922	0.063	0.015	0.000	3.3	6.8	5.1
ABS-MCF20-CNT1	0.865	0.130	0.005	0.000	4.4	5.8	5.1
ABS-MCF20-CNT2	0.859	0.131	0.011	0.000	6.0	7.4	6.7
ABS-MCF20-CNT3	0.852	0.132	0.016	0.000	7.5	8.9	8.2
ABS-MCF30-CNT1	0.790	0.204	0.006	0.000	5.8	7.9	6.8
ABS-MCF30-CNT2	0.783	0.205	0.011	0.000	7.4	9.5	8.4
ABS-MCF30-CNT3	0.777	0.207	0.017	0.000	8.9	11.0	10.0
ABS-MCF10-GNP3	0.921	0.063	0.000	0.016	20.2	27.1	23.6
ABS-MCF10-GNP6	0.903	0.064	0.000	0.033	38.9	52.1	45.5
ABS-MCF10-GNP9	0.885	0.065	0.000	0.050	57.7	77.1	67.4
ABS-MCF20-GNP3	0.852	0.131	0.000	0.017	21.6	29.2	25.4
ABS-MCF20-GNP6	0.832	0.134	0.000	0.034	40.3	54.2	47.2
ABS-MCF20-GNP9	0.812	0.136	0.000	0.052	59.1	79.2	69.1
ABS-MCF30-GNP3	0.776	0.206	0.000	0.018	23.0	31.3	27.1
ABS-MCF30-GNP6	0.754	0.210	0.000	0.036	41.7	56.3	49.0
ABS-MCF30-GNP9	0.732	0.214	0.000	0.054	60.5	81,.3	70.9

Table S2. Average melt flow index (MFI) at 10 kg and 220 °C of ABS multiscale composites (from CM samples).

Type of composite	Avg. MFI (g/10 min)
CM_ABS	33.1 ± 1.2
CM_ABS-MCF10	25.6 ± 1.9
CM_ABS-MCF20	20.8 ± 0.8
CM_ABS-MCF30	17.4 ± 1.4
CM_ABS-MCF10-CNT1	15.3 ± 1.0
CM_ABS-MCF10-CNT2	8.1 ± 0.9
CM_ABS-MCF10-CNT3	2.7 ± 0.2
CM_ABS-MCF20-CNT1	10.9 ± 0.6
CM_ABS-MCF20-CNT2	4.5 ± 0.3
CM_ABS-MCF20-CNT3	1.2 ± 0.1
CM_ABS-MCF30-CNT1	6.5 ± 0.5
CM_ABS-MCF30-CNT2	3.2 ± 0.2
CM_ABS-MCF30-CNT3	0.5 ± 0.0
CM_ABS-MCF10-GNP3	20.3 ± 1.4
CM_ABS-MCF10-GNP6	15.1 ± 0.9
CM_ABS-MCF10-GNP9	11.7 ± 0.4
CM_ABS-MCF20-GNP3	15.3 ± 0.8
CM_ABS-MCF20-GNP6	9.3 ± 0.5
CM_ABS-MCF20-GNP9	7.4 ± 0.4
CM_ABS-MCF30-GNP3	9.8 ± 0.5
CM_ABS-MCF30-GNP6	6.3 ± 0.5
CM_ABS-MCF30-GNP9	5.3 ± 0.3

Another parameter P2 = $P_{E,\,MFI,\,H}$ that maximizes both the stiffness, the processability, and the hardness can be calculated from Equation (S1):

$$P_{E,MFI,H} = P_{E,MFI} \times H = E_T \times MFI \times H \quad (S1)$$

where E_T, MFI, and H represent the tensile modulus, the melt flow index, and the hardness (micro).

Specific data are reported in **Table 5**, **Table S2**, and **Table S3**.

Table S3. Results of nanoindentation tests of ABS multiscale CM samples. The results of Elastic modulus (En) and Hardness (H) are reported as an average of 300 measurements. Selection parameter P2=$P_{E, MFI, H}$ is also reported according to Equation S1. In column P2, the bold values evidence the selected compositions for FFF.

Type of composite	Avg. E_n (GPa)	Avg. H (GPa)	P2= $P_{E,MFI, H}$ × 10^{-3} (GPa^2 g/10min)
CM_ABS	2.84 ± 0.12	0.16 ± 0.02	**12.3**
CM_ABS-MCF10	3.25 ± 0.48	0.19 ± 0.08	18.3
CM_ABS-MCF20	3.71 ± 0.55	0.18 ± 0.08	**22.5**
CM_ABS-MCF30	4.44 ± 0.78	0.22 ± 0.10	25.2
CM_ABS-MCF10-CNT1	3.58 ± 0.61	0.21 ± 0.10	**11.6**
CM_ABS-MCF10-CNT2	3.29 ± 0.44	0.17 ± 0.06	5.31
CM_ABS-MCF10-CNT3	3.57 ± 0.60	0.21 ± 0.11	2.22
CM_ABS-MCF20-CNT1	4.00 ± 0.84	0.24 ± 0.17	**13.5**
CM_ABS-MCF20-CNT2	3.98 ± 0.74	0.24 ± 0.15	5.73
CM_ABS-MCF20-CNT3	4.15 ± 0.78	0.25 ± 0.18	**1.60**
CM_ABS-MCF30-CNT1	4.78 ± 0.97	0.31 ± 0.21	**12.9**
CM_ABS-MCF30-CNT2	4.55 ± 0.95	0.31 ± 0.21	6.32
CM_ABS-MCF30-CNT3	4.76 ± 1.03	0.28 ± 0.18	8.03
CM_ABS-MCF10-GNP3	3.86 ± 0.55	0.18 ± 0.04	13.4
CM_ABS-MCF10-GNP6	4.03 ± 0.48	0.19 ± 0.04	11.6
CM_ABS-MCF10-GNP9	3.93 ± 0.59	0.16 ± 0.07	8.91
CM_ABS-MCF20-GNP3	4.26 ± 0.62	0.22 ± 0.12	**20.5**
CM_ABS-MCF20-GNP6	4.34 ± 0.75	0.19 ± 0.09	**10.8**
CM_ABS-MCF20-GNP9	4.47 ± 0.78	0.20 ± 0.12	9.48
CM_ABS-MCF30-GNP3	4.66 ± 0.83	0.26 ± 0.15	**18.4**
CM_ABS-MCF30-GNP6	5.23 ± 1.01	0.25 ± 0.13	13.0
CM_ABS-MCF30-GNP9	5.53 ± 0.91	0.23 ± 0.13	11.2

In order to consider stiffness, processability and conductivity, the selective parameter, P3 = $P_{E,MFI,Res,}$ has been recently proposed for the comparison of the filler effects [38] , and it is defined by Equation (S2):

$$P_{E,MFI,Res} = P_{E,MFI}/Res = E_T \times MFI / Res \qquad (S2)$$

where Res is the resistivity. The higher the $P_{E,MFI,Res,}$ the better the properties, the higher the desirability of the composition for processability and properties. According to this parameter as expected all the composites with CNT exhibited the higher desirability parameters (see **Table S4**).

Table S4. Electrical resistivity (Res) of ABS multiscale CM samples as a function of the applied voltage. Selection parameter P3=$P_{E,MFI,Res}$ is also reported according to Equation (S2).

Type of composite	Voltage (V)	Res (Ω.cm)	Avg. Res (Ω.cm)	P3= E_T × MFI/Res (GPa^2 g/10min Ω^{-1} · cm^{-1})
CM_ABS	100	$(1.7 \pm 0.4)\times10^{15}$	$(1.7 \pm 0.4)\times10^{15}$	4.50 ×1 0^{-11}
CM_ABS-MCF10	100	$(1.4 \pm 0.5)\times10^{15}$	$(1.4 \pm 0.5)\times10^{15}$	6.86×10^{-11}
CM_ABS-MCF20	100	$(3.9 \pm 0.3)\times10^{14}$	$(3.9 \pm 0.3)\times10^{14}$	3.2×10^{-10}
CM_ABS-MCF30	5	$(8.1 \pm 1.4)\times10^{8}$	$(8.1 \pm 1.4)\times10^{8}$	1.41×10^{-4}
CM_ABS-MCF10-CNT1	2	17.5 ± 5.3	17.7 ± 5.4	3.12×10^{3}
	5	17.7 ± 5.5		
	12	17.8 ± 5.5		
	24	17.8 ± 5.5		
	30	17.5 ± 5.2		
CM_ABS-MCF10-CNT2	2	7.2 ± 1.5	7.3 ± 1.5	4.28×10^{3}
	5	7.2 ± 1.6		
	12	7.5 ± 1.3		
CM_ABS-MCF10-CNT3	2	2.1 ± 0.6	2.0 ± 0.6	5.28×10^{3}
	5	2.0 ± 0.6		
	12	2.0 ± 0.7		
CM_ABS-MCF20-CNT1	2	6.5 ± 0.4	6.5 ± 0.3	8.66×10^{3}
	5	6.5 ± 0.2		
	12	6.5 ± 0.2		
CM_ABS-MCF20-CNT2	2	2.0 ± 0.4	1.9 ± 0.4	12.6×10^{3}
	5	1.9 ± 0.3		
	12	1.9 ± 0.3		
CM_ABS-MCF20-CNT3	2	1.5 ± 0.2	1.5 ± 0.2	4.27×10^{3}
	5	1.5 ± 0.2		
	12	1.5 ± 0.2		
CM_ABS-MCF30-CNT1	2	5.4 ± 0.9	5.4 ± 0.9	7.69×10^{3}
	5	5.3 ± 0.8		
	12	5.4 ± 0.9		
CM_ABS-MCF30-CNT2	2	1.2 ± 0.2	1.2 ± 0.2	17.0×10^{3}
	5	1.2 ± 0.2		
	12	1.2 ± 0.2		
CM_ABS-MCF30-CNT3	2	0.7 ± 0.2	0.7 ± 0.2	4.10×10^{3}
	5	0.7 ± 0.2		
	12	0.7 ± 0.2		
CM_ABS-MCF10-GNP3	100	$(7.7 \pm 3.3)\times10^{9}$	$(7.7 \pm 3.3)\times10^{9}$	9.64×10^{-6}
CM_ABS-MCF10-GNP6	100	$(3.1 \pm 0.7)\times10^{8}$	$(3.1 \pm 0.7)\times10^{8}$	1.96×10^{-5}
CM_ABS-MCF10-GNP9	100	$(1.3 \pm 0.4)\times10^{7}$	$(1.3 \pm 0.4)\times10^{7}$	4.28×10^{-5}
CM_ABS-MCF20-GNP3	100	$(9.8 \pm 0.4)\times10^{8}$	$(9.8 \pm 0.4)\times10^{8}$	9.5×10^{-6}
CM_ABS-MCF20-GNP6	100	$(8.7 \pm 7.1)\times10^{7}$	$(8.7 \pm 7.1)\times10^{7}$	6.53×10^{-6}
CM_ABS-MCF20-GNP9	100	$(6.6 \pm 3.5)\times10^{7}$	$(6.6 \pm 3.5)\times10^{7}$	7.18×10^{-6}

CM_ABS-MCF30-GNP3	2	346 ± 260	397 ± 277	175
	5	324 ± 178		
	12	403 ± 269		
	24	483 ± 382		
	30	427 ± 294		
CM_ABS-MCF30-GNP6	2	43 ± 20	60 ± 32	811
	5	68 ± 37		
	12	64 ± 34		
	24	64 ± 36		
	30	62 ± 34		
CM_ABS-MCF30-GNP9	2	342 ± 140	382 ± 150	121
	5	399 ± 148		
	12	403 ± 160		
	24	394 ± 155		
	30	373 ± 149		

Table S5. Electrical resistivity (Res) of ABS multiscale 3D-printed samples as a function of the applied voltage. Comparative resistivity ratio between 3D-printed and compression-molded samples.

Type of composite	Voltage (V)	Res (Ω.cm)	Avg. Res (Ω.cm)	Resistivity ratio 3D vs. CM
3D_ABS-MCF20-CNT1	2	13.5 ± 2.1	13.7 ± 2.7	2.10
	5	14.0 ± 3.1		
	12	13.9 ± 2.6		
	24	13.6 ± 2.7		
	30	13.4 ± 2.8		
3D_ABS-MCF20-CNT3	2	3.9 ± 1.0	3.9 ± 1.0	2.57
	5	3.9 ± 1.0		
	12	3.8 ± 1.0		
	24	3.9 ± 1.0		
	30	3.8 ± 1.1		
3D_ABS-MCF30-CNT1	2	1.6 ± 0.3	1.6 ± 0.2	0.30
	5	1.7 ± 0.3		
	12	1.6 ± 0.1		
	24	1.6 ± 0.1		
	30	1.6 ± 0.2		
